# Hybrid XGBoost-RF-MLP model and PSO optimization for performance and emissions of CI engine using waste cooking biodiesel blends

**DOI:** 10.1038/s41598-025-29269-8

**Published:** 2025-12-12

**Authors:** M. S. Gad, M. Sami Soliman, Emad B. Helal

**Affiliations:** 1 Mechanical Engineering Department, Faculty of Engineering, Fayoum University, Fayoum, Egypt; 2 Department of Seismology, National Research Institute of Astronomy and Geophysics (NRIAG), Helwan, 11421 Egypt; 3 Department of Cyber Security, College of Engineering, Almaqaal University, Basraa, 61014 Iraq

**Keywords:** Performance: emissions, WCO, XGBoost, Random forest, MLP, Energy science and technology, Engineering

## Abstract

Transesterification was used to create methyl ester from waste cooking oil (WCO). Diesel oil and biodiesel blends in 25, 50, 75, and 100% were developed and authorized by ASTM. The primary contribution of this study lies in integrating experimental WCO biodiesel data with a novel hybrid machine learning and Particle Swarm Optimization (PSO) framework. A hybrid model, combining XGBoost, Random Forest, and MLP, was developed to predict engine performance and emissions. The core novelty is the use of base model predictions as meta-features for a final meta-learner, createing a superior stacked ensemble. This hybrid model was then coupled with PSO to identify optimal engine operating conditions. Key experimental results revealed that pure biodiesel (B100) reduced CO, HC, and smoke emissions by 25%, 43%, and 45%, respectively. However, increased NOx emissions by 23% and brake-specific fuel consumption by 22% were shown compared to diesel at full load. Crucially, the hybrid model demonstrated exceptional predictive accuracy, achieving a significantly lower Mean Squared Error (MSE in the order of 10⁻⁷) across all 13 output parameters compared to the individual MLP (MSE ~ 10⁻^3^), RF (MSE ~ 10⁻⁴), and XGBoost (MSE ~ 10⁻⁶) models. The PSO algorithm successfully converged to an optimal solution of 86% engine load and 26% biodiesel blend (B26), maximizing the defined fitness function that balanced performance and emissions. The results unequivocally demonstrate that the proposed hybrid modeling approach offers a robust and highly accurate framework for engine optimization, establishing WCO biodiesel as a viable alternative fuel when used in optimal blends.

## Introduction

The Earth suffers from the negative environmental effects of fossil fuels use. The release of greenhouse gases, such carbon dioxide, contributes to major environmental problems, including climate change and adverse effects on the economy and ecology. By reducing carbon emissions, the Sustainable Development Goals (SDGs) aim to save the environment and advance the development of sustainable alternative fuels as biodiesel^1^. However, it is challenging to promote biodiesel as a fuel despite its advantages, with production costs, fuel properties, and feedstock availability being vital obstacles to decreasing the demand for pure diesel. Hybrid intelligent models have played a crucial role in enhancing the prediction accuracy of engine performance and emissions. Singh et al.^2^ combined Adaptive Neuro-Fuzzy Inference System (ANFIS) with Genetic Algorithm (GA), showing improved estimations of brake thermal efficiency (BTE), hydrocarbons (HC), and nitrogen oxides (NOx). While this hybridization improved accuracy, it required careful GA tuning, and limiting scalability. Extending this work, Singh et al.^3^ applied the Grasshopper Optimization Algorithm (GOA) to optimize diesel–biodiesel–ethanol blends, confirming its effectiveness at specific blend ratios but exposing sensitivity to input composition. Veza et al.^4^ and Shirneshan et al.^5^ employed response surface methodology (RSM) and Box–Behnken design (BBD), identifying optimal parameters with < 7% error. These statistical approaches offered interpretability but were restricted to fixed operating ranges. Collectively, these early studies reveal the trade-off between algorithmic accuracy and model adaptability.

Recent work has integrated optimization with neural networks. Samuel et al.^6^ developed an improved particle swarm optimization with back propagation neural network (IMPSO-BPNN) for hydrogen–natural gas mixtures, achieving a mean absolute percentage error (MAPE) of 0.771%, significantly outperforming GA-BPNN, GA-SVM, and PSO-SVM. However, its complexity raised concerns over computational feasibility. Ramachander et al.^7^ applied Gaussian regression to diesel injection systems, reporting correlation coefficients near unity but requiring high-quality datasets. Simsek et al.^8^ confirmed that kernel-based extreme learning machine (K-ELM) provided more stable and generalizable results compared to LS-SVM and PSO, though interpretability was limited. Similar efforts by Bitire and Jen^9^ and Said et al.^10^ using GRNN-PSO achieved accurate emission predictions, but remained highly parameter-sensitive. Statistical design methods persisted in parallel, with Ardebili et al.^11^ and Manimaran et al.^12^ applied RSM and CCD to biodiesel blends, offered accurate yet narrow-scope predictions. Together, these studies underscore the contrast between high-accuracy, complex AI models and interpretable but rigid statistical methods.

Kumar and Pal^13^ refined RSM-based optimization for algal biodiesel with fuel additives, achievied < 6.5% error but with limited adaptability. ANN applications gained prominence with Can et al.^14^ and Hosamani et al.^15^, who confirmed ANN’s predictive reliability (R^2^ near 1) while emphasized its black-box nature. Advanced integrations by Esonye et al.^16^ and Zheng et al.^17^ with optimization algorithms improved predictive strength but demanded high computational resources. Earlier work by Shivakumar et al.^18^ validated ANN’s adaptability in variable compression ratio engines, though calibration dependency persisted. Rajkumar et al.^19^ combined ANN with genetic algorithms and combustion modeling, offered a balance of accuracy and interpretability. Duan et al.^20^ developed IMPSO-BPNN with near-perfect correlation coefficients, but its tuning complexity raised concerns about transferability. These works collectively mark a shift from statistical models to ANN-based hybrids, improving accuracy but exacerbating transparency and data dependency issues.

To overcome these, Zandie et al.^21^ developed multi-input, multi-output ML models for diesel–gasoline–biodiesel blends, demonstrating robustness under variable loads but requiring dense experimental data. Maheshwari et al.^22^ emphasized nonlinear regression for multi-objective optimization, reliable but functionally rigid. Tosun et al.^23^ showed ANN outperforming linear regression for biodiesel–alcohol blends, though interpretability remained problematic. Bendu et al.^24^ applied GRNN–PSO with ethanol-fueled HCCI engines, achieving accuracy but with parameter sensitivity. Wong et al.^25,26^ confirmed the value of quadratic prediction models in scarce datasets, highlighting efficiency but also overfitting risks. Newer algorithms such as Extreme Learning Machine (ELM) were tested by Santhosh et al.^27^, who achieved rapid convergence with ZnO nanoparticle blends, though stability under noisy data was questioned. Sebayang et al.^28^ compared ANN and ELM for Sterculia foetida biodiesel, finding ELM superior in emission prediction yet less robust across blends. Collectively these references ^21–28^, highlight the rise of faster, more efficient ML methods (ELM, GRNN–PSO, hybrid ANN), but emphasize persistent interpretability and robustness challenges.

Aghbashlo et al.^29^ integrated ELM with wavelet transforms (ELM-WT), enhancing accuracy with low RMSE. Wong et al.^30^ and Silitonga et al.^31^ confirmed K-ELM’s superiority over RBFNN and LS-SVM, though with increased complexity. Kusumo et al.^32^ and Ghanbari et al.^33^ used kernel-based methods and genetic programming (GP), achieving high R^2^ but facing reproducibility challenges. Alruqi et al.^34^ applied Bayesian-optimized Gaussian processes, improving reliability but at computational cost. Sharma et al.^35^ leveraged Taguchi and utility theory for biodiesel–diesel blends, offering straightforward optimization but limited treatment of nonlinear interactions. Together with literature ^29–35^, reveal a turn toward kernel and probabilistic models that trade interpretability for accuracy and generalizability. Ensemble methods also gained traction. Poompipatpong^36^ validated quadratic models in marine engines, cost-effective but narrow in scope. Sharma and Sahoo^37^ demonstrated that boosted regression trees (BRT) outperform ANN in both precision and interpretability, signaling the potential of ensemble learning. Ghanbari et al.^38^ reinforced the utility of RSM for nanoparticle-diesel blends, though again limited in scope. Foundational works by Huang et al.^39^ and Ding et al.^40^ outlined ELM’s theoretical advantages but warned against instability under real-world uncertainty. These findings suggest ensemble and kernel-based methods as emerging alternatives to ANN dominance.

Comparative assessments strengthened this narrative. Sahin^41^ found ANN superior for BTE and NOx prediction, SVM for exhaust temperature, and extreme gradient boosting best for CO_2_ and HC. Viswanathan et al.^42^ and Tan et al.^43^ confirmed ANN’s superiority over RSM, though both remained reliable. Chaki and Biswas^44^ enhanced ANN with entropy–FA optimization, reducing errors to ~ 1.75% but retaining interpretability concerns. Wang et al.^45^ integrated GA-SVM with NSGA-III, delivering strong generalization at the expense of computational simplicity. Said et al.^46^ validated Gaussian regression for dual-fuel diesel–biogas engines, reporting near-perfect R^2^. Hasanzadeh et al.^47^ showed RSM reliable with ~ 5% error. Broader reviews by Sharma^48^ and Aliramezani et al.^49^ positioned AI-based prognostic modeling as the future of CI engine optimization, noting that ensemble and hybrid ML methods outperform conventional approaches but remain constrained by dataset dependency and interpretability.

The performance and exhaust emissions of a diesel engine powered by a blend of diesel fuel and waste oil biodiesel were anticipated and optimized using model structures created with Artificial Neural Networks (ANN) and Response Surface Methodology (RSM). The correlation coefficient (R^2^) for each response in the developed model ranged from 0.9785 to 0.9997. An ANN model with a maximum mean absolute error of 1.723% and *R* > 0.99 was employed to predict all responses. The RSM approach returned a desirability value of 0.750, indicating satisfactory performance. To analyze RSM regression equations, we employed the Actor Critic with the Kronecker-Factored Trust Region-Differential Evolution (ACKTR-DE) and Harris Hawks Optimization (HHO) algorithms^50^. An artificial neural network (ANN) model was provided to estimate the emissions and performance of various biodiesel percentages as engine speed varies. All variables had correlation coefficients greater than 0.99 and R^2^ values higher than 0.98. MSE, MAPE, and MSLE values were low but had a substantial predictive ability^51^. Two mathematical models as extreme learning machine (ELM) and quadratic regression were used to forecast engine characteristics and emissions at varying engine speeds and biodiesel concentrations. Quadratic regression outperformed ELM in forecasting engine performance and emissions for the majority of factors, resulting in reduced root-mean square and mean absolute percentage errors^52^.

Particle toxicity and tiny particles (less than 23 nm are more damaging). This effect is particularly obvious during vehicle cold-start operation, which is an unavoidable daily driving scenario in which after-treatment systems malfunction. The data showed that as the engine warms up, PN increases for all fuels while particle size decreases. The PN concentration in a fully warmed-up engine was up to 132% higher than in a cold start. Particles of 23 nm accounted for a substantial proportion of PN (9%) but only 0.1% of PM. During cold start, a fuel blend containing 5% lubricating oil boosted PN concentration while decreasing particle size^53^. Most automobiles require a cold start as part of their normal operation. The engine warm-up time was divided into seven parts: formal hot-start and cold-start, as well as intervals that are not classed as cold-start or hot-start under regulations. The results showed that as the engine warmed up, the fuel exergy, exhaust heat losses, and exergy destruction were decreased by 2.3, 34.1, and 34.1%, respectively, while the exhaust exergy loss was increased by 43.5%. As the engine warmed up, the FMEP and BSFC were decreased by 56.7% and 14.9%, respectively, while the BTE and exergetic efficiency was increased by 5.6% and 5.3%^54^.

Random Forests were used to choose input variables, while PSO and GA were used to establish the optimal model hyperparameters. Hybrid models perform well in both training and validation datasets, with R values greater than 0.980 and 0.937, respectively. All R^2^ values are greater than 0.930, indicating excellent generalization. Hybrid models effectively address the limitations of single algorithms and are the best approach for applying machine learning to emission prediction^55^. Six machine learning regression models, Decision Tree (DT), Random Forest (RF), Gradient Boosting (GB), Extra Trees (ET), Extreme Gradient Boosting (XGB), and Light GBM, were trained to forecast five critical outputs: brake thermal efficiency, brake specific fuel consumption, carbon dioxide, particulate matter, and nitrogen oxides. GB outperformed RF and XGB in terms of predictive performance, with average R^2^ values of 0.999 (train) and 0.9586 (test) and MAPE of 2.58%^56^. Engine behavior was modeled and predicted using artificial neural network (ANN) and machine learning (ML) approaches. The R^2^ values of the model showed exceptional agreement with experimental data, exceeding 0.93 for BSFC, 0.97 for EGT, and 0.98 for NOx and HC, indicating outstanding predictive capacity across all parameters^57^.

While numerous studies have applied individual or hybrid models, there is a distinct lack of research that employs a stacked ensemble framework which uses the predictions of multiple, diverse base models as inputs to a superior meta-learner specifically for modeling engines fueled with Waste Cooking Oil (WCO) biodiesel. Furthermore, a truly integrated approach that uses such a high-fidelity model as a digital twin for PSO to discover optimal engine settings remains unexplored for this application. Previously, studies used a single machine learning model (e.g., ANN, RSM, ANFIS) or compared models side by side. Some investigations use simple model averaging. This model demonstrates a stacked hybrid modeling architecture. A two-level stacked ensemble was built. At the outset, three fundamentally different models (MLP, XGBoost, and Random Forest) were trained separately. XGBoost can handle complex feature interactions. Random Forest is used for robustness and low over fitting. MLP captures deep nonlinear interactions, boosting prediction accuracy and generalization compared to single models. Their predictions were then used as fresh input features (meta-features) for a second-level meta-learner (another XGBoost model) that learnt how to combine them optimally. This architecture enables the meta-learner to identify the exact scenarios in which each base model works optimally. For example, it may learn to trust Random Forest’s prediction for braking power more than MLP’s, although XGBoost’s prediction for CO emission may be weighted more heavily. This advanced error-correction process goes beyond a basic model comparison or average, yielding to a much lower MSE (10^−7^ vs. 10^−3^ for MLP). Conventional optimization (e.g., RSM) is frequently limited to basic, predefined polynomial relationships. Other studies employ PSO to tune model hyper parameters. The trained hybrid model serves as a high-fidelity alternative for the real engine. The PSO method searches for the best load and fuel blend combination by querying this rapid, accurate surrogate model within the fitness function, rather of relying on expensive physical trials or less accurate individual models. This results in a powerful closed-loop system. This allows for the virtual investigation of millions of alternative operating points, identifying the global optimum (load = 0.86, blend percentage = 0.26), which would be impractical to uncover by testing alone. The fitness function weights were set to represent a realistic trade-off between performance and emissions. For small datasets such as (25 points), tree-based models outperform standard neural networks (MLPs). The poor performance of the standalone MLP (highest MSE) demonstrates the dangers of using a complicated model prone to over fitting on minimal data. The improved performance of XGBoost and hybrid model shows that gradient boosting and stacking generalization are better paradigms for this type of problem.

The main uniqueness of this work is the establishment of an integrated framework rather than the implementation of well-established individual models. This framework includes (1) stacked ensemble architecture for improved prediction accuracy on small datasets, and (2) closed-loop optimization in which the PSO algorithm searches for the optimal (load, biodiesel blend ratio) combination by querying the hybrid model as a fast, accurate digital twin, rather than relying on expensive physical experiments or less accurate individual models.

Therefore, the objective of this study is to develop and validate a novel integrated framework that combines a stacked hybrid machine learning model with PSO optimization for a diesel engine running on WCO biodiesel blends. The specific aims are:

1. To develop a stacked ensemble model using XGBoost, Random Forest, and MLP as base learners. The XGBoost algorithm was selected as the meta-learner due to its built-in L1 and L2 regularization that prevents over fitting and its powerful gradient boosting framework that optimally combines complex, non-linear predictions from the base models.
2. To integrate this hybrid model with a PSO algorithm, configured with a swarm size of 100 and 100 iterations to ensure robust exploration and convergence in the two-dimensional search space, alongside established cognitive and social parameters to identify the optimal combination of engine load and biodiesel blend ratio.
3. To comprehensively evaluate the framework’s accuracy against experimental data, analyze why the ensemble approach reduces errors compared to individual models like the MLP, and elucidate the performance-emission trade-offs of WCO biodiesel to provide practical insights for engine calibration and blend design.

This work shows a comparative evaluation of three modeling techniques as random forest, XGBoost and MLP models. Hybrid modeling combines the effects of three modeling techniques. The accuracy of the prediction model was shown by comparing the outputs of the modeling approach with the experimental findings. The engine’s performance was evaluated using the following metrics: brake power, mean effective pressure, exhaust gas temperature, thermal efficiency, fuel-air ratio, equivalence ratio, volumetric efficiency, and specific fuel consumption. Studies have been conducted on exhaust concentrations, including smoke, CO, HC, and NOx. Combining ensemble learning (XGBoost-RF) with deep learning (MLP) and PSO optimization would result in much higher predictive accuracy than independent models. The hybrid PSO-ML framework can efficiently generalize to previously unknown biodiesel blend ratios, resulting in reliable projections for engine performance and emission trends. Optimized hybrid ML models can replace experimental testing in biodiesel engine studies, saving money and time.

## Methodology

### Biodiesel production

Although diverse feedstocks such as soybean, palm, and jatropha oils have been widely used in biodiesel production, waste cooking oil (WCO) has specific economic, environmental, and sustainability benefits that make it an ideal choice for large-scale biodiesel production. WCO is a low-cost, widely available feedstock produced in vast quantities by the home and commercial food processing sectors. Its use greatly lowers biodiesel manufacturing costs, which are otherwise driven by the cost of virgin oils. The valorization of WCO solves environmental and waste management issues. The improper dumping of spent cooking oil into sewage systems results in substantial water contamination and environmental destruction. Converting this garbage into biodiesel is a circular economy strategy that transforms a problematic waste stream into a profitable renewable energy supply. WCO-derived biodiesel lowers lifecycle greenhouse gas emissions. The feedstock does not compete with food resources, which aligns with the United Nations’ Sustainable Development Goals (SDGs) for responsible consumerism and climate action. As a result, WCO is chosen for its abundance, cost-effectiveness, waste-to-energy potential, and contribution to environmental sustainability, making it an ideal feedstock for creating predictive hybrid models for biodiesel engine applications.

**Fig. 1:**
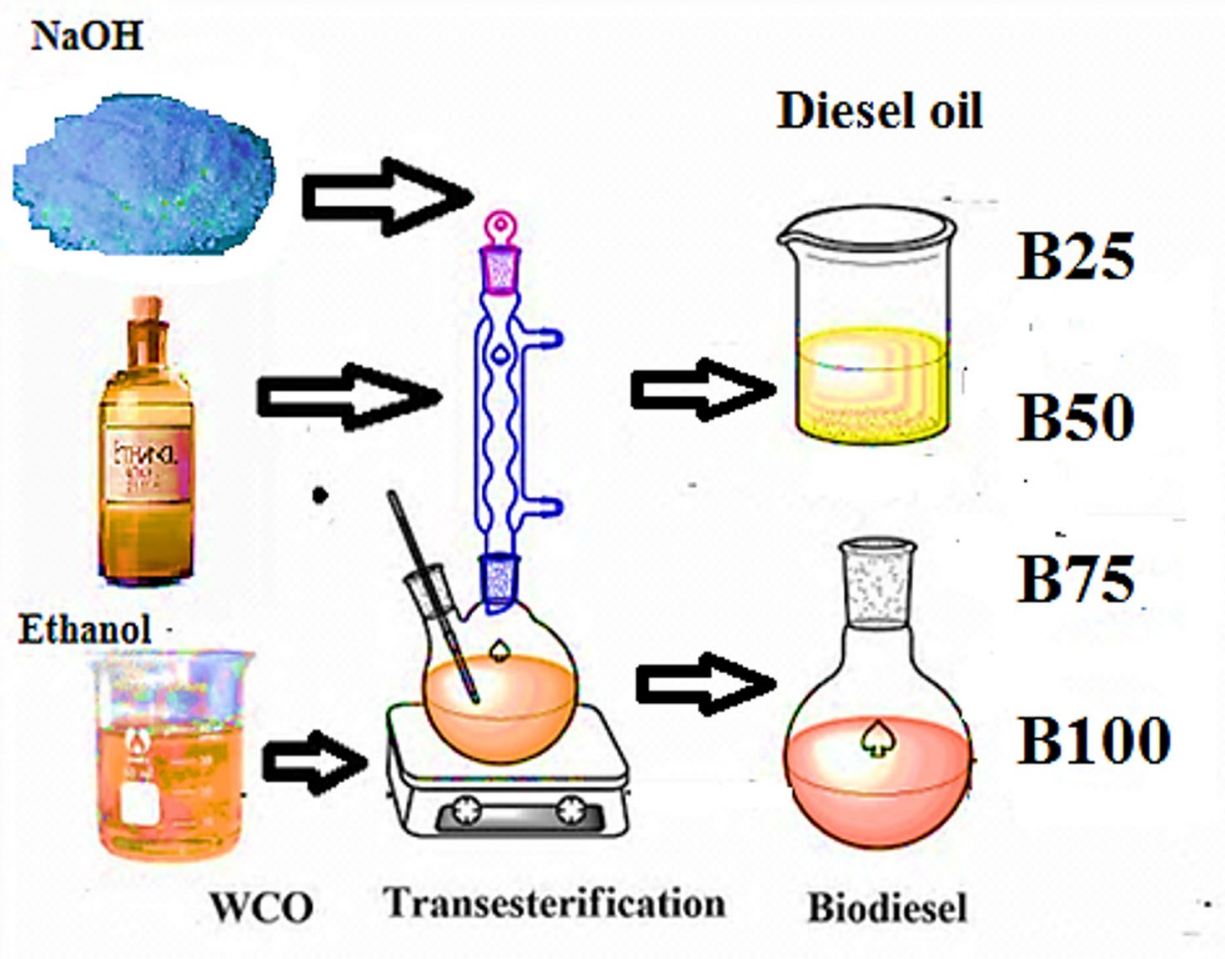
Biodiesel production and its blends preparation.

WCO from restaurants and hotels was filtered to get rid of impurities and gums. Due to its increased viscosity, WCO is not utilized in direct way in diesel engines. During transesterification, WCO was changed into methyl ester. WCO was preheated to 110 °C and filtered to remove moisture. The oil was then transferred into a flask that was held up by a magnetic stirrer, thermometer and condenser. Methoxide was produced by mixing 1:9 molar methanol with 1.5% by weight NaOH. The mixture of oil and methoxide was stirred for 90 min at 60 °C to produce glycerin and methyl ester. The glycerin and ester were extracted by leaving the mixture in the separating funnel for 12 hrs. Warm water was used to remove the catalyst, unreacted methanol and contaminants. A rotary evaporator was used to dry the biodiesel once the water has been removed to produce crude methyl ester. Pure diesel was combined with methyl ester at volume ratios of 0, 25, 50, 75, and 100%. Figure 1 depicts the manufacturing of biodiesel and the creation of its blends. Table 1 lists the properties of crude diesel and methyl ester mixtures.

The authorization or approval of biodiesel blends depends on national and international fuel standards that specify allowable mixing ratios and fuel properties according to ASTM (American Society for Testing and Materials) Standards. ASTM D-445, ASTM D-93, ASTM D-4052, ASTM D-224 and ASTM D-13 specify parameters such as viscosity, flash point, specific gravity, calorific value and cetane number, respectively. ASTM D7467 covers blends of biodiesel up to 20% biodiesel. ASTM D6751 describes biodiesel specifications.

**Table 1:** WCO biodiesel and its blends’ properties.

Properties	Method	WCO Biodiesel (B100)	Diesel oil
Kinematic viscosity @40 °C, cSt	ASTM D-445	4.7	3
Flash point, °C	ASTM D-93	122	74
Specific gravity @15 °C	ASTM D-4052	0.885	0.837
Lower heating value, MJ/kg	ASTM D-224	39.6	42.1
Cetane number	ASTM D-13	53	50

### Experimental test rig

**Fig. 2:**
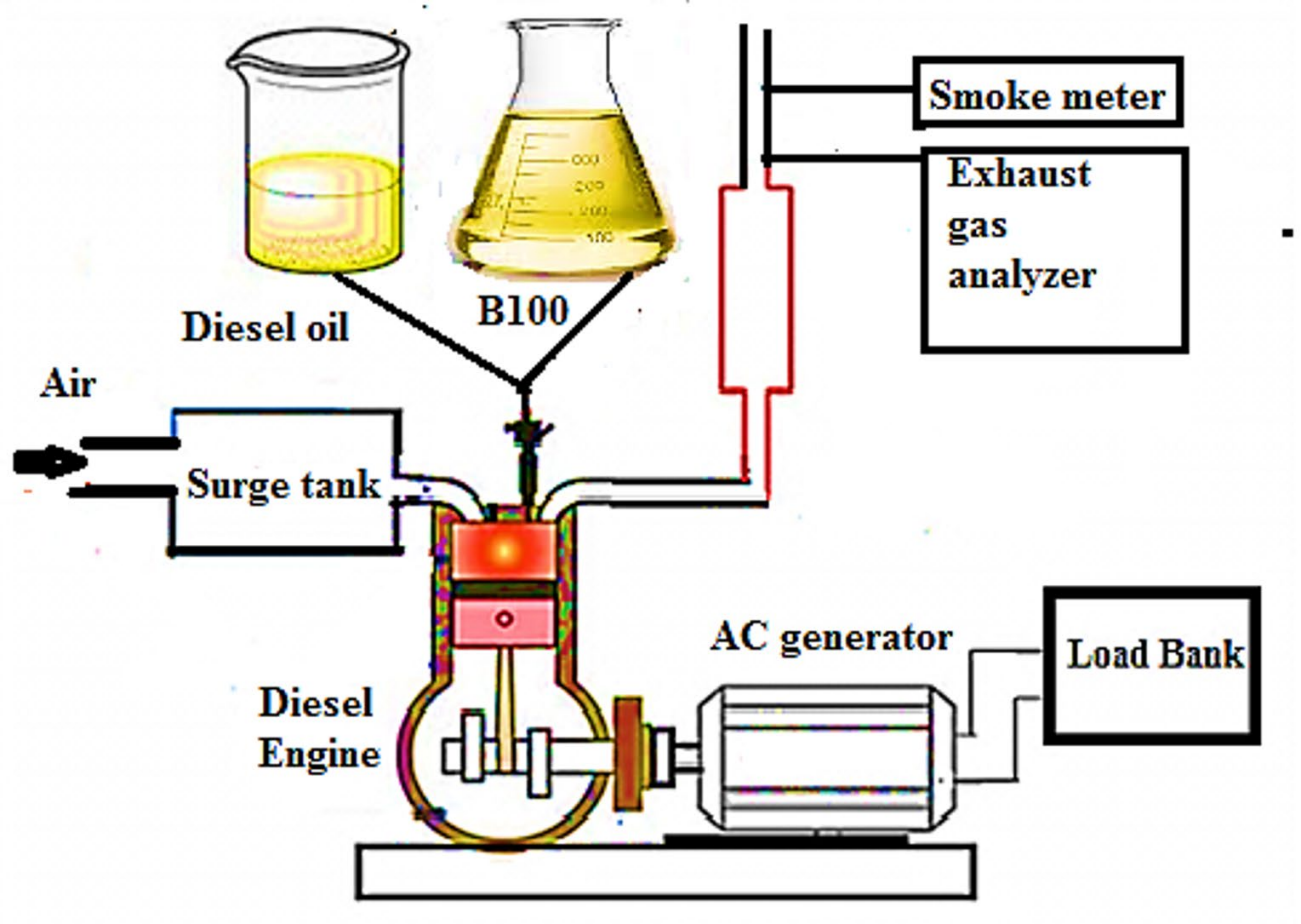
Schematic diagram of experimental setup.

The experiment used a four-stroke, air-cooled, diesel engine that could provide up to 10 kW of power at 1500 rpm. Bore of engine is 100 mm, its stroke is 105 mm, and its compression ratio is 17.5:1. Figure 2 shows the setup schematic diagram. The engine’s output power was measured by connecting an AC generator with an electrical output of 15 kW directly to the engine. Output voltage and current data were used to determine how much electricity the load bank consumed. To assess the intake air flow, a sharp edge orifice was placed at the air box’s side to reduce the pulsing air flow. The pressure decrease across the orifice was tracked using U-tube manometer. The intake air and exhaust gas temperatures were measured using Type K thermocouple. Measurements of carbon monoxide, NO_x_, smoke and HC exhaust gases were conducted. A gas analyzer (O_2_ (0–22%) electrochemical cell, NO_2_ (0–1000 ppm) electrochemical), NO (0–4000 ppm) electrochemical cell, CO_2_ (0–10%) NDIR bench, HC (0–2000 ppm) NDIR bench, and smoke meter (opacity 0–99% and resolution 0.1) were utilized.

Before the tests, the engine was first run without load for 20 min in order to warm it up using diesel oil under steady state conditions. After engine stabilization, the readings were collected. The engine running continuously at 1500 rpm and with a range of engine loads was used for each measurement. To guarantee that the testing results were reliable and reproducible, each engine test condition was done three times with identical operating parameters. To minimize random experimental variance, the measured values were averaged over three replicates. The mean values were presented in the results section, and the standard deviation of each set of measurements was calculated and used to construct error bars in the accompanying figures. This statistical technique gives a quantitative measure of variability and increases the level of confidence in the presented patterns. Prior to testing, all measurement instruments were carefully calibrated in accordance with the manufacturer’s specifications to ensure the experimental data’s accuracy and repeatability. In terms of thermal efficiency, hydrocarbons, Exhaust gas analyser was calibrated using approved span gases with known amounts of CO, CO₂, HC, and NOₓ. Prior to each test session, zero calibration was performed using pure nitrogen. The smoke meter was calibrated using the manufacturer’s standard reference filters to check the opacity scale. NOx, carbon monoxide, and smoke emissions, the uncertain ranges were ± 1 ppm, ± 1 ppm, ± 0.01% vol., ± 1%, and ± 1.5%, respectively. Engine speed, brake power, exhaust gas temperature, and specific fuel consumption were determined to have the highest measurement errors, at 0.2, 0.85, 0.2, 2.2, and 0.15%, respectively. By summing up all of the parameter uncertainties, the overall uncertainty was calculated using the following formula.

$$\begin{gathered} \:\sqrt {\left( {uTexh} \right)^{2} + \left( {ubp} \right)^{2} + \left( {usfc} \right)^{2} + \left( {uN} \right)^{2} + \left( {uther} \right)^{2} + \left( {uCO} \right)^{2} + \left( {uHC} \right)^{2} + \left( {uNOx} \right)^{2} } \hfill \\ = \:\sqrt {\left( {0.2} \right)^{2} + \left( {0.85} \right)^{2} + \left( {2.2} \right)^{2} + \left( {0.15} \right)^{2} + \left( {1.5} \right)^{2} + \left( {0.01} \right)^{2} + \left( 1 \right)^{2} + \left( 1 \right)^{2} + \:\left( {0.2} \right)^{2} + \left( 1 \right)^{2} \:} \hfill \\ = \: \pm \:2.9\% \hfill \\ \end{gathered}$$

where:

Uncertainties of output power (ubp), EGT (uTexh), engine speed (uN), CO concentration (uCO), HC emission (uHC), BSFC (usfc), BTE (uther), and NOx (uNOx).

### Modeling of emissions and engine performance

Based on the collection of experimental input-output data, this model employs various combinations of engine loads and fuel blends. Three mathematical models as XGBoost regression, Random Forest regression (RF), and Multi-layer Perceptron ANN (MLP) were used to forecast the output variables.

#### Data collection and preprocessing

The dataset includes two primary features, load and Fuel, and thirteen target variables representing various performance metrics: Brake power (kW), Mean effective pressure (bar), BSFC (kg/kW h), BTE (%), Fuel-air equivalence ratio, EGT (°C), Air-fuel ratio, Volumetric efficiency (%), CO (ppm), CO_2_ (%), HC (ppm), NOx (ppm), and Smoke emissions (%). In data preprocessing stage, the input for machine learning models was normalized to a range^1^. These, multiple target variables were predicted using the following machine learning model. Three groups were created from the dataset: training, testing, and validation. Training data is considered 90% of the original data while testing and validation have similar split of 5% of the original data. To avoid explicit train-validation-test divides, an approach of using the complete dataset for both validation and testing was used due to data limitations. The specified parameters, such as engine load and biodiesel blend ratio, have the greatest impact on engine performance and exhaust emissions. These parameters are experimentally controllable and physically meaningful, allowing for accurate modeling and real-world optimization of diesel engines.

#### System models

**Fig. 3:**
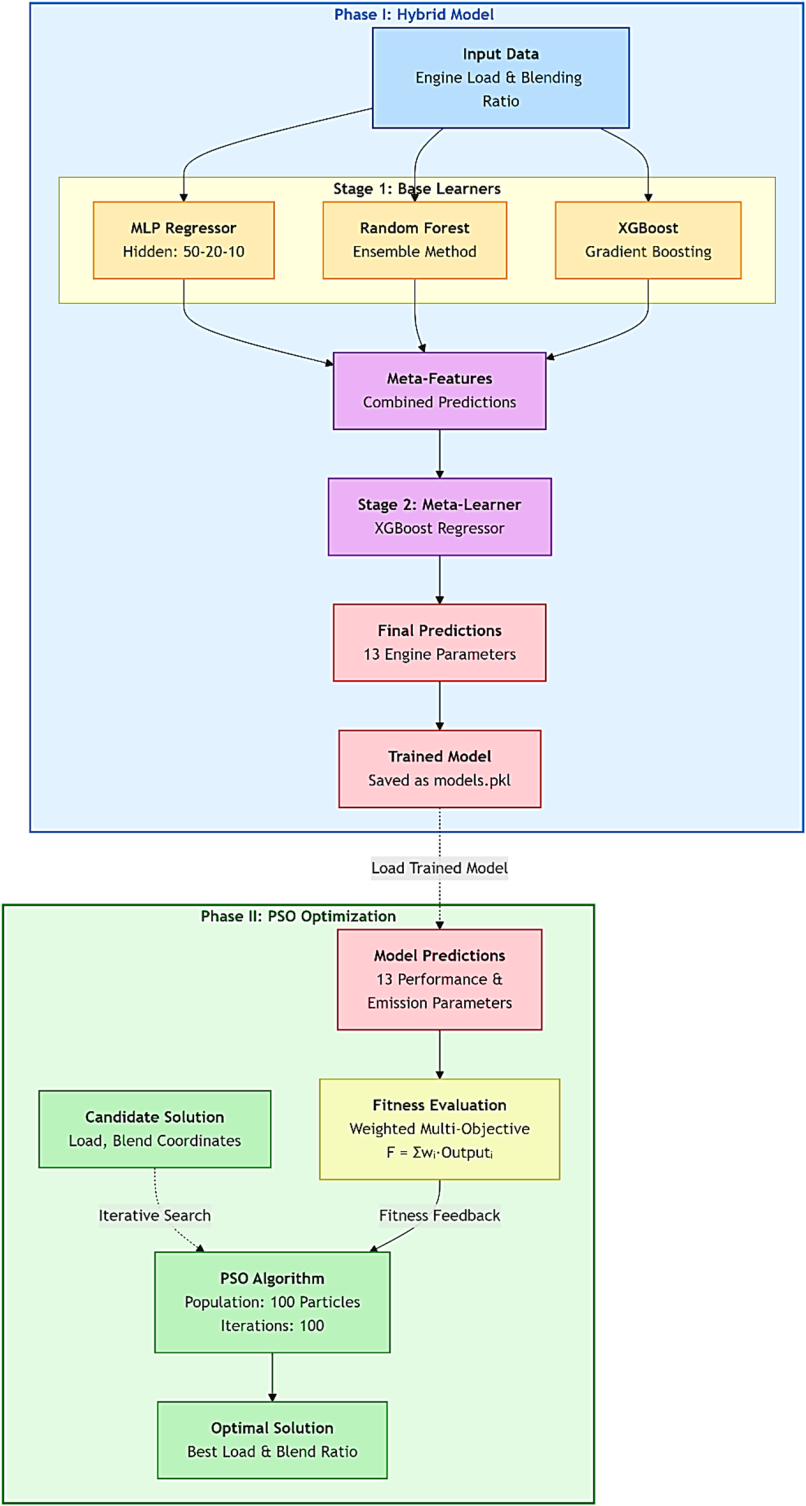
Hybrid stacking ensemble and PSO optimization framework, showing the two-stage prediction model (left) and operational optimization process (right).

The proposed approach combines Extreme Gradient Boosting (XGBoost), Random Forest (RF), and Multilayer Perceptron (MLP) into a two-stage hybrid framework. Stage 1 (Base Learners): These models effectively capture nonlinear interactions and feature relevance in the dataset. Stage 2 (XGBoost Meta-Learner): The predictions from all three base models (XGBoost, RF, and MLP) are combined to form meta-features that serve as input to the XGBoost meta-learner. The meta-learner refines these predictions by learning complex residual patterns, which increases overall accuracy and generalization. The Particle Swarm Optimization (PSO) algorithm is subsequently employed to identify optimal engine operating conditions that balance performance and emissions. The methodology for system modeling is as shown in Fig. 3.

For the initial predictions, three machine learning models were chosen: Random Forest Regressor^58,59^, XGBoost Regressor^60,61^, and Multi-Layer Perceptron (MLP) Regressor^62,63^. MLP uses several hidden layers (50-20-10 neurons) to capture intricate, non-linear relationships within data. The neural network’s adaptability allows the model to cope with a variety of input-output mappings, making it appropriate for challenging regression issues. XGBoost is a version of boosting decision trees recognized for its rapid performance and superior precision. It employs a boosting methodology, incrementally incorporating weak learners to reduce error, which allows it to effectively capture complicated relationships among features. By constructing numerous decision trees during training, ensemble learning method known as random forest produces the average prediction for regression problems. It provides information on feature relevance while reducing model variance.

Subsequently, stacking and aggregation stage were implemented. This Stacked Ensemble Model is composed of predictions from the three base models (MLP, XGBoost, and Random Forest). The stacking model applied the XGBoost Regressor as the meta-learner to leverage the meta-features for final prediction. The selection was driven by XGBoost’s ability to effectively prevent overfitting and handle complex relationships in the feature space. Through the integration of several models’ strengths, the stacked model seeks to improve the overall prediction accuracy. Stacked Ensemble Modeling combines the advantages of multiple models to improve forecast precision. The meta-model is trained using the predictions from these underlying models as input features (meta-features). The concept is that the meta-model identifies and rectifies the errors of the base models, utilizing their collective strengths to generate more precise predictions for all 13 engine performance and emission parameters.

##### XGBoost modeling

Regression problems can be solved quickly and accurately using the sophisticated machine learning algorithm XGBoost. This method of ensemble learning involves building a sequence of decision trees, each of which aims to fix the mistakes of the one before it. The prediction accuracy of model is improved by this recurring boosting technique. When it comes to handling complex, non-linear relationships in data, XGBoost excels. Regularization settings, which penalize model complexity, are part of the strategy to prevent overfitting. It ensures that every tree in the model is less affected by noise in the training data and more concentrated on improving performance. Particularly in competitive data science jobs, XGBoost performs better than rival regression methods. It is the preferred choice for large-scale regression problems due to its speed, accuracy, and scalability, which offers significant advantages in predictive modeling tasks^60,61^.

##### Random forest modeling

For regression situations, random forest is a reliable and flexible ensemble learning technique. In order to increase forecast accuracy and manage overfitting, it creates a large number of decision trees during training and aggregates their outputs. Using a method called bootstrap aggregation or bagging, each decision tree in the forest has been trained on a random subset of the data, both in terms of features and samples. Random Forest produces reliable predictions even with noisy data since it combines the results of several trees in regression. Large datasets with numerous variables and intricate relationships are especially well-suited for Random Forests. To describe nonlinear linkages and interactions, the method doesn’t require a lot of parameter tweaking^58,59^.

##### Multi-layer perceptron (MLP) model

An artificial neural network with several layers of neurons, or nodes, arranged hierarchically is called Multi-Layer Perceptron (MLP). It is one of the most often used and simple types of neural networks, particularly for supervised learning tasks like classification and regression. Nonlinear activation functions are typically used by the MLP’s neurons, allowing the network to identify complex input patterns. MLPs are crucial models for machine learning tasks like pattern recognition, regression, and classification because they can identify nonlinear relationships in data. One kind of feed forward neural network made up of completely linked neurons with a nonlinear activation function is called a multilayer perceptron. The MLP model’s inferior performance can be due to its sensitivity to hyper parameter adjustment and small dataset size, which may have resulted in local minima and overfitting difficulties. It is frequently used to separate nonlinearly separable data^62,63^.

##### Hybrid model

The hybrid model increases the accuracy of predictions regarding output variables in engine performance analysis by combining the benefits of three specific machine learning techniques: Random Forest, XGBoost (XGB), and Multi-Layer Perceptron (MLP). This hybrid approach, as opposed to individual models, makes use of each technique’s distinct advantages. By integrating both approaches, the hybrid model improves on their combined strengths and dramatically reduces prediction mistakes like Mean Squared Error (MSE). The hybrid model is especially useful for predicting emissions and engine performance indicators in complex engine systems because of its integration, which ensures increased precision and dependability. This hybrid stacking strategy delivers better generalization and lower MSE than any single model.

### Particle swarm optimization (PSO)

Once individual model predictions have been generated, the weighting strategy and parameters of model outputs aggregating are optimized using Particle Swarm Optimization (PSO)^64,65^. PSO was used because it can balance exploitation (personal experience) with exploration (social impact) to discover the best answers. PSO serves two primary purposes in this work:

1. Hyperparameter optimization: Particle Swarm Optimization (PSO) optimizes each model’s hyperparameters to successfully lower prediction errors by viewing the ensemble model as a search domain.
2. Meta-model enhancement: PSO improves the weights assigned to each model’s outputs, enabling flexible, data-driven prediction integration. Managing complex interactions and making sure the ensemble approach can adapt to different datasets.

The PSO algorithm was used for optimization, with hyperparameters carefully chosen to balance convergence speed and solution quality. The population size and maximum iteration number were chosen to give adequate search variety while minimizing computing expense. The weight was reduced linearly in order to guarantee a smooth transition from exploration to exploitation. The acceleration coefficients were chosen using widely accepted ranges from previous optimization studies resulting in stable convergence behavior. These parameters were initially tuned through a series of preliminary trials, which revealed that smaller or larger values slowed convergence or resulted in premature local optima. The selected setup provided the greatest consistent prediction accuracy (lowest MSE) across numerous runs of the hybrid PSO-ML models. In the parameter space, which is defined by two important variables, engine load and blending ratio, each of the initialized 100 particles in the swarm represents a possible solution. Initial velocities and locations are assigned at random within defined boundaries. To assess each particle’s location based on the stacked ensemble models’ prediction quality, a fitness function is created. Fitness of each output is estimated by calculating the weighted total of several goal variables as follows:

$$\begin{gathered} \:Fitness = w_{1} \cdot \:Brake\:Power + w_{2} \cdot \:mean\:effectie\:pressure \hfill \\ - w_{3} \cdot \:Specific\:Fuel\:Consumption + w_{4} \cdot \:{\text{Thermal}}\:{\text{efficiency}} \hfill \\ + w_{5} \cdot \:Fuel\:air\:equivalence\:ratio + w_{6} \:\: \cdot \:\:Exhaust\:Gas\:Temperature \hfill \\ + w_{7} \cdot \:Air\:Fuel\:Ratio + w_{8} \cdot \:Volumetric\:Efficiency \hfill \\ + w_{9} \cdot \:CO\:Emission + w_{{10}} \cdot \:CO2\:Emission \hfill \\ + w_{{11}} \cdot \:HC\:Emission + w_{{12}} \cdot \:NOx\:Emission \hfill \\ + w_{{13}} \cdot \:Smoke\:Emission \hfill \\ \end{gathered}$$

where $\:{w}_{i}$ is weight coefficients for each target variable $\:i$ ($\:where\:i=1,\:2,\:3,\dots\:,\:12$), calculating out each variable’s proportionate significance in the fitness score. Depending on how each component should affect total fitness, these weights might be zero, positive, or negative. The following criterion is used to choose the weight values^64–66^:

$$\:w\:=\:\left[0.3,\:0,\:-0.3,\:0.3,\:0,\:0,\:0,\:0,\:-0.01,\:-0.03,\:-0.01,\:-0.04,\:-0.01\right]$$

The goal is to get maximum prediction performance by maximizing the fitness function. In PSO, the fitness function evaluates each solution (set of weights and hyper parameters) based on the ensemble’s prediction error rate. Maintaining a mixture between local and global search capabilities, PSO updates particle locations and velocities based on local and global bests as it iterates toward an optimal solution.

These weights are not arbitrary. It were chosen to represent a logical engineering objective that balances desirable and undesirable outcomes. Positive weights (+ 0.3) were used to calculate brake power and thermal efficiency: parameters. It was utilized to increase engine power and fuel economy. These are the major performance indicators for all engine applications. Negative weights (− 0.3) were employed to reduce specific fuel consumption, which has a direct influence on operational costs and the environment. To reduce harmful emissions, negative weights for CO and NOx were utilized (− 0.01 and − 0.04, respectively). The varying magnitudes represent a prioritization. The larger weight for NOx (− 0.04) indicates that it is a critical pollutant with strict regulatory restrictions, giving its removal a higher priority in the optimization. Zero weights (e.g., mean effective pressure) were employed. These parameters were determined to have a significant correlation with other, already-weighted factors, hence biasing the fitness function without adding new information. In summary, the weights represent a specific optimization goal: to discover the operating condition that delivers the optimal balance of high power, high efficiency, and low emissions, with a significant emphasis on minimizing NOx emissions. This is a typical case for engine calibration under modern environmental rules.

## Results and discussion

### Brake power (BP)

**Fig. 4:**
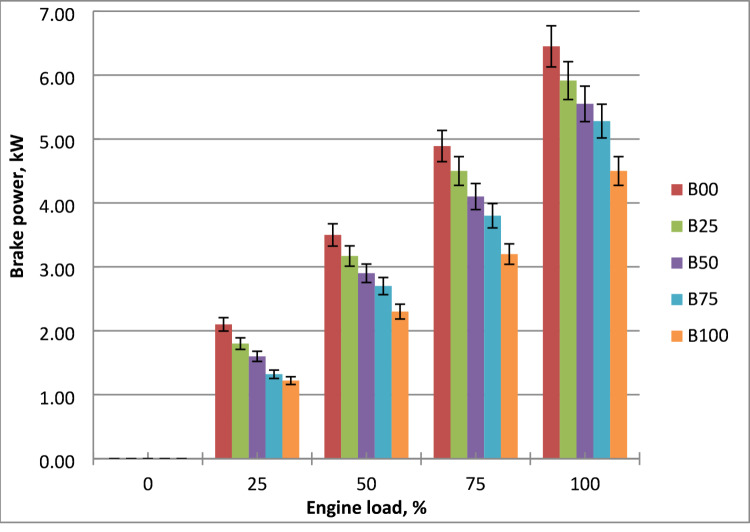
Output power of the fuels at engine load variation.

Impact of biodiesel mixtures on engine output power is depicted in Fig. 4. It is clear that brake power rises in tandem with engine load. Increased methyl ester content reduces calorific value, leading to lower output power despite higher fuel consumption at increased loads. Because of its greater density and viscosity, methyl ester demonstrated worse fuel atomization, fuel-air mixing, and vaporization. Biodiesel contains oxygen (about 10–12 wt%) in its molecular structure, lowering its heating value by 10–15% compared to diesel. As a result, less energy is released per unit of fuel, resulting in a lower power output. Biodiesel generates less power than diesel oil due to its lower calorific value. For the same amount of power, biodiesel and other diesel oil blends require more fuel. The lowest output power of biodiesel at 1500 rpm is 23% less than diesel oil under maximum load. The results were agreed with references^9,12,15^.

### Mean effective pressure (MEP)

**Fig. 5:**
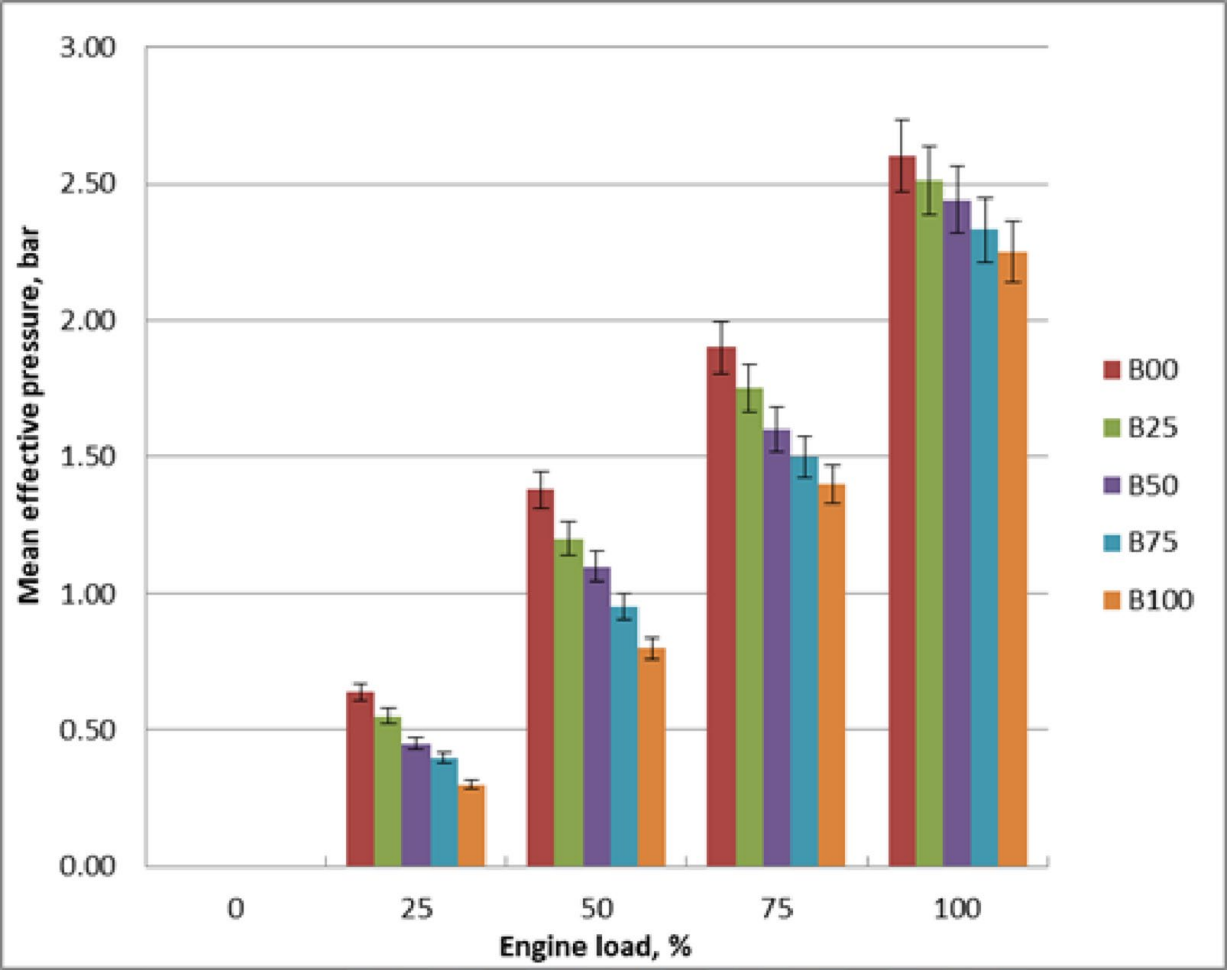
Mean effective pressure of tested fuels at loads changes.

The impact of biodiesel blends on engine mean effective pressure is depicted in Fig. 5. It is clear that mean effective pressure rises in tandem with engine load. Fuel consumption rises as engine load increases. MEP decreases as a result of methyl ester’s decreased calorific value brought on by an increase in its proportion. The increased viscosity of biodiesel has an effect on atomization and spray penetration and results in bigger droplets and poorer mixing with air, particularly at partial loads. This results in less efficient combustion. The methyl ester hinders fuel- air mixing, atomization and vaporization due to its higher density and viscosity. Pure diesel and biodiesel blends need more fuel to produce the same amount of power because methyl ester has a lower calorific value. MEP of B100 is 23% lower than diesel oil at 1500 rpm and peak load. The findings were agreed with literature^9,10,12^.

### Brake specific fuel consumption (BSFC)

**Fig. 6:**
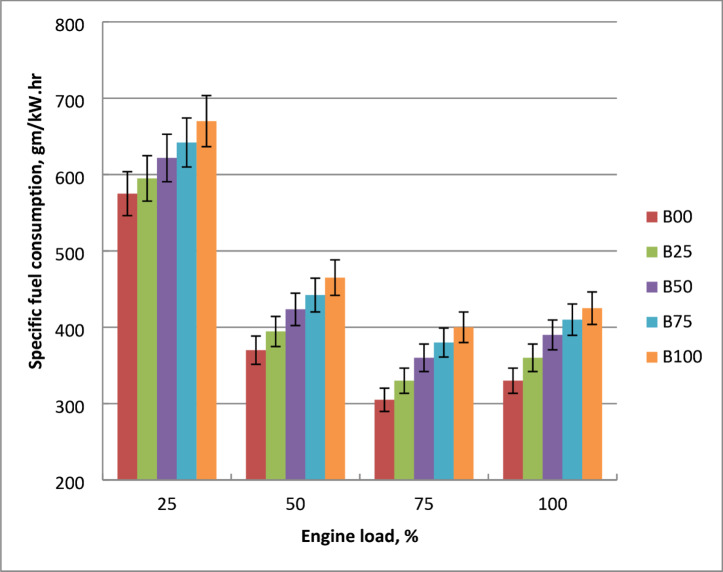
BSFC for tested blends at loads variations.

Figure 6 shows how engine output power affects the precise fuel consumption of blends of methyl ester and pure diesel. Diesel oil requires less fuel than biodiesel for all output power range. The engine needs more fuel to produce the same amount of power for methyl ester than diesel fuel. As engine load increased, BSFC values fell for both fuels. Because methyl ester has lower calorific value about pure diesel, its BSFC is higher than crude diesel. The higher density of biodiesel results in more mass flow for the same injection time. Furthermore, higher viscosity results in less effective atomization and air-fuel mixing, lowering combustion efficiency and necessitating more fuel to maintain the same load. These properties of methyl ester lead to problems with air-fuel mixing, vaporization, and atomization. Biodiesel’s worse combustion, decreased volatility, and increased viscosity are the primary causes of its higher BSFC. Molecular frictional forces of biodiesel are the causes of elevated BSFC. When compared to diesel oil, biodiesel had the greatest BSFC of 22% at engine full load. The results were confirmed with references^9,10,15^.

### Brake thermal efficiency (BTE)

**Fig. 7:**
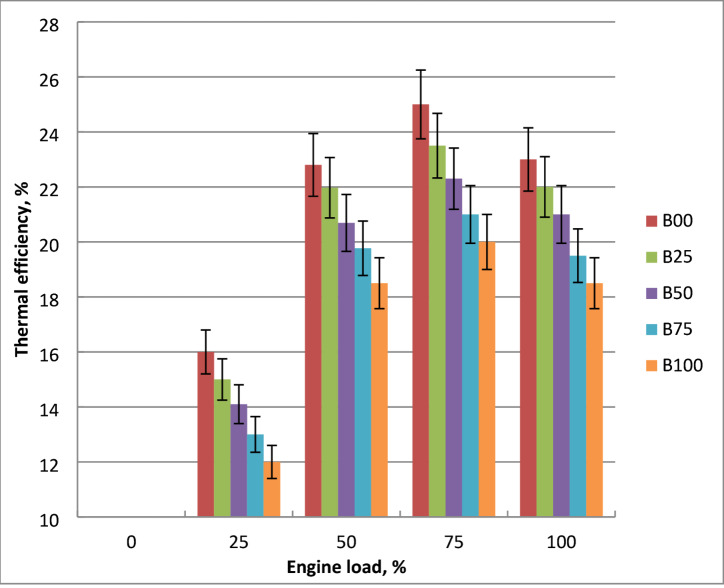
Impact of tested fuel on BTE with engine brake power variation.

The difference in BTE when utilizing diesel and biodiesel mixtures is seen in Fig. 7. Fuel consumption rises in tandem with engine load. The thermal efficiency decreases as the percentage of methyl ester increases. BTE peaks and then starts to decrease as engine output power rises. Lower engine brake power leads to more concentrated fuel use and higher heat loss. Because of increased fuel consumption and friction losses, the rise in engine output power results in higher BSFC. Biodiesel has reduced thermal efficiency due to its increased viscosity, poor combustion characteristics, and low volatility. Biodiesel’s increased surface tension cause bigger droplets and less homogenous air-fuel mixtures. This results in incomplete combustion and less effective heat release, reducing thermal efficiency. Atomization, vaporization and air- fuel mixing problems are shown due to these properties of biodiesel. This decline in BTE would be explained by methyl ester’s reduced lower heating value and volatility when compared to crude diesel. Higher engine output power result in higher heat loss and fuel consumption. At maximum load, methyl ester’s BTE drops by 21% in comparison to diesel fuel. The literature validated these findings^7,21,23^.

### Exhaust gas temperature (EGT)

**Fig. 8:**
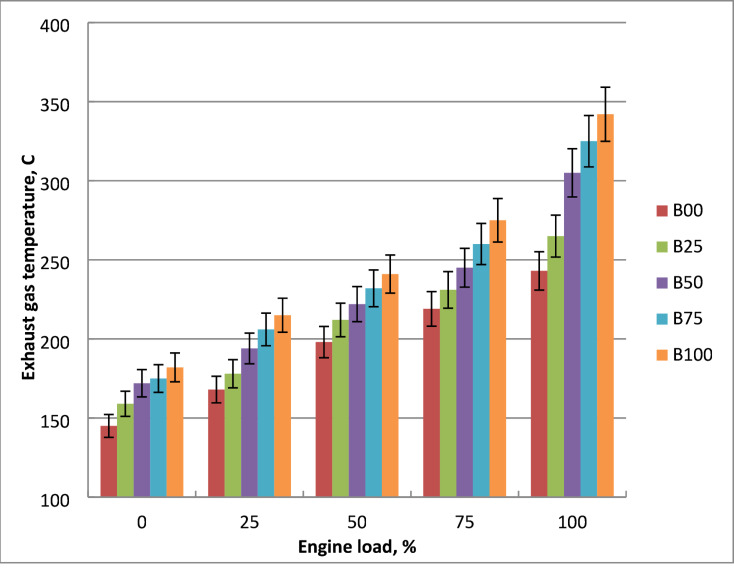
EGT of the used blends at engine output variation.

Figure 8 shows the relationship between engine power and exhaust gas temperature for methyl ester and diesel mixtures. As engine load rises, the temperature of exhaust gases rises for all fuels. This could happen because higher load causes the engine to use more fuel, which raises the cylinder temperature and increases exhaust gases heat loss. Because more of the generated heat exits the cylinder with the exhaust gases rather than being turned into usable work, EGT rises even while brake thermal efficiency falls. Because diesel-methyl ester mixtures burn and heat up less efficiently than diesel fuel, their EGT are greater at the load variation. The reduced volatility and higher viscosity of biodiesel causes issues with atomization and vaporization, which raises the EGT of methyl ester about crude diesel. In comparison to pure diesel, methyl ester has lower calorific value and more heat loss, which lowers the BTE and raises the EGT. These features of biodiesel cause issues with vaporization, atomization, and air-fuel mixing. In comparison to diesel oil, EGT of B100 increased by 28% at highest output load. These results were corroborated by the literature^7,17,19^.

### Air-Fuel ratio (A/F)

**Fig. 9:**
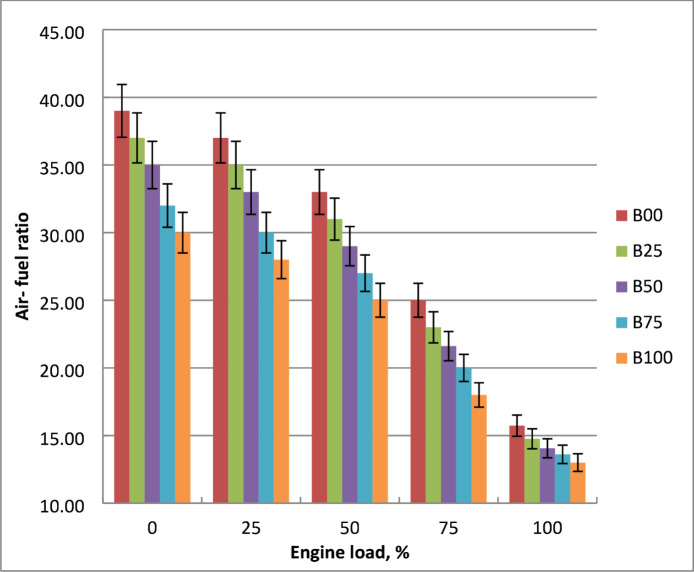
Impact of tested fuel on air-fuel ratio with engine brake power variation.

Figure 9 illustrates how the air-fuel ratio for diesel and methyl ester blends is impacted by engine brake power. Air-fuel ratio decreases as engine load rises. A/F should be lowered since a higher engine load produced higher fuel flow rate. Diesel fuel uses less fuel than methyl ester blends and has a larger calorific value, therefore biodiesel fuel has the declined air-fuel ratio. Methyl ester blends have lower stoichiometric A/F than diesel. Because biodiesel mixtures construction of 11% more O_2_ about diesel, they require less air to run. Fuel consumption is increased by the density and viscosity of methyl ester. These features of biodiesel cause issues with vaporization, atomization, and air-fuel mixing. In comparison to diesel, biodiesel mixtures have lower stoichiometric A/F ratio. The amount of fuel utilized for a given volume rises when diesel and biodiesel are blended, while the actual air-fuel ratio falls. At 100% engine load, the methyl ester fuel-air ratio was decreased by 13% about diesel oil. The findings were agreed with references^9,12,15^.

### Fuel-air equivalence ratio

**Fig. 10:**
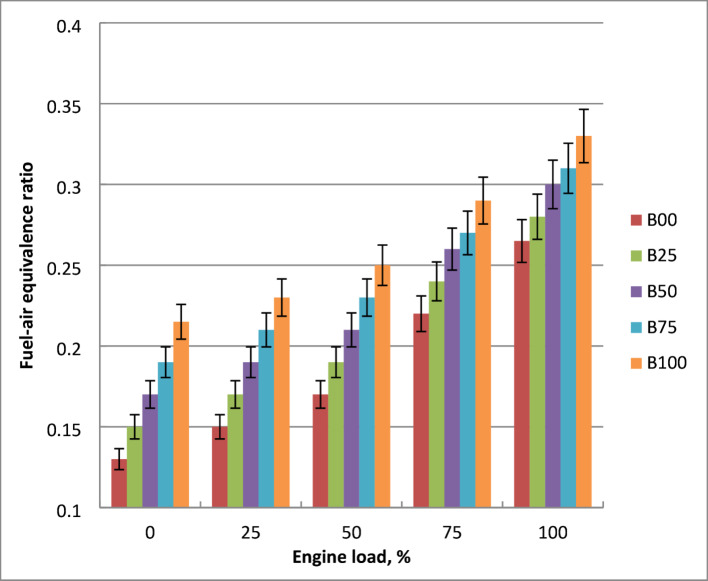
Effect of biodiesel blends on fuel- air equivalence ratio.

Figure 10 indicates how the output power affects the fuel-air equivalence ratio for diesel and biodiesel mixtures. As engine load increases, the equivalence ratio climbs. Since a higher engine load causes a higher fuel flow rate, the equivalence ratio should be adjusted. Diesel fuel burns less fuel and has larger calorific value than methyl ester blends, hence methyl ester fuel has a lower equivalence ratio. Biodiesel mixtures have lower stoichiometric A/F than pure diesel. Blends of biodiesel require less air to run since they contain 11% more oxygen than diesel. Methyl ester’s density and viscosity rise as the fuel flow rate does. Higher density requires more fuel per cycle, and its oxygenated structure naturally requires less external air for complete combustion than diesel. These characteristics of biodiesel lead to problems with air-fuel mixing, atomization and vaporization. Related to diesel oil, methyl ester mixtures have lower stoichiometric A/F. Actual air-fuel ratio falls and the amount of fuel utilized for a given volume rises when diesel and biodiesel are combined. At peak engine load, the equivalence ratio of biodiesel was decreased by 14% about crude diesel. The literature validated these results^9,21,23^.

### Volumetric efficiency

**Fig. 11:**
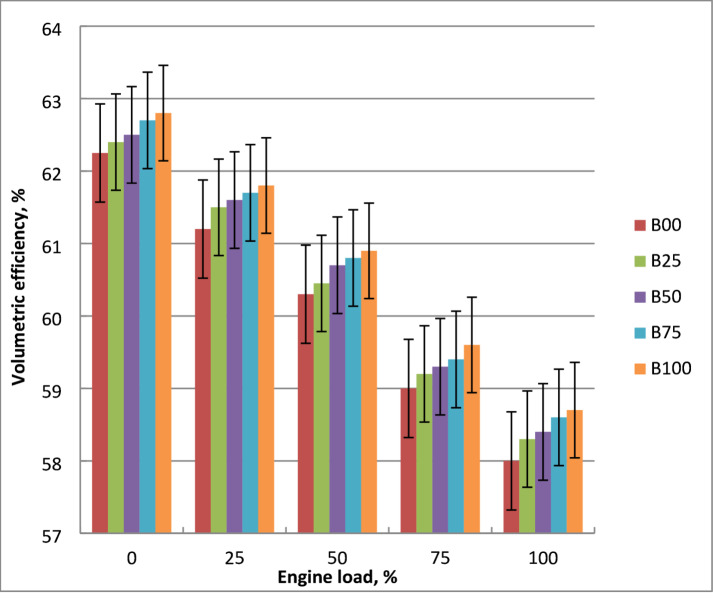
Volumetric efficiency of tested fuels at different loads.

The difference in volumetric efficiency between diesel and methyl ester blends as a function of engine load is shown in Fig. 11. It decreases because of the restrictions of air flow in intake manifold at higher engine loads. Volumetric efficiency is significantly impacted by engine load because of the higher residual gas temperature. At increasing engine output power, it drops due to severe air throttling brought on by constrained airflow in the intake air. Volumetric efficiency of methyl ester mixtures with larger methyl ester content is lower. Exhaust gas temperatures of biodiesel mixtures are higher than diesel. Methyl ester fuel burns with less air since it includes 11% oxygen. Biodiesel’s evaporative cooling effect, particularly at partial loads, can reduce intake air temperature and increase air density, leading to improved volumetric efficiency. Due to variations in their thermal characteristics and latent heat of vaporization, biodiesel has greater cylinder temperature and lower input air temperature. Crude diesel oil has 10% greater volumetric efficiency than B100 at highest brake power. The findings were validated with literature^7,12,23^.

### CO emissions

**Fig. 12:**
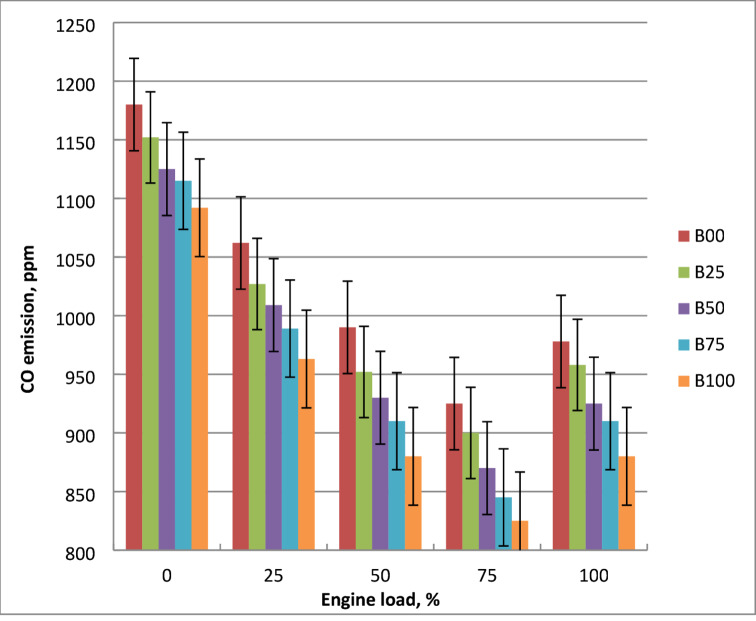
Effect of methyl esters on CO concentrations at engine load change.

As shown in Fig. 12, engine output power affects CO levels of diesel and biodiesel mixtures. When engine output power increases, CO emissions begin to rise after initially declining to a minimum. Engine load affects the quantity of carbon monoxide generated because lower engine output power promotes slower rates of CO oxidation by reducing gas cylinder temperatures. Lower gas cylinder temperatures at lower engine loads promote the slow rate of CO oxidation. Compared to crude diesel, methyl ester emits less carbon monoxide. Oxygen-rich molecular structure of biodiesel facilitates improved combustion and reduces the likelihood of the creation of fuel-rich zones. Biodiesel has a lower carbon-to-hydrogen ratio than diesel, which means that less carbon is accessible for CO production per unit of fuel burned. Because methyl ester contains more oxygen than diesel oil, it has been demonstrated to have improved combustion efficiency and air-fuel mixing. At maximum output power, B100 reduces CO emissions by 25% in comparison to diesel fuel. Previous investigations have shown similar results^15,17,21^.

### CO_2_ emission

**Fig. 13:**
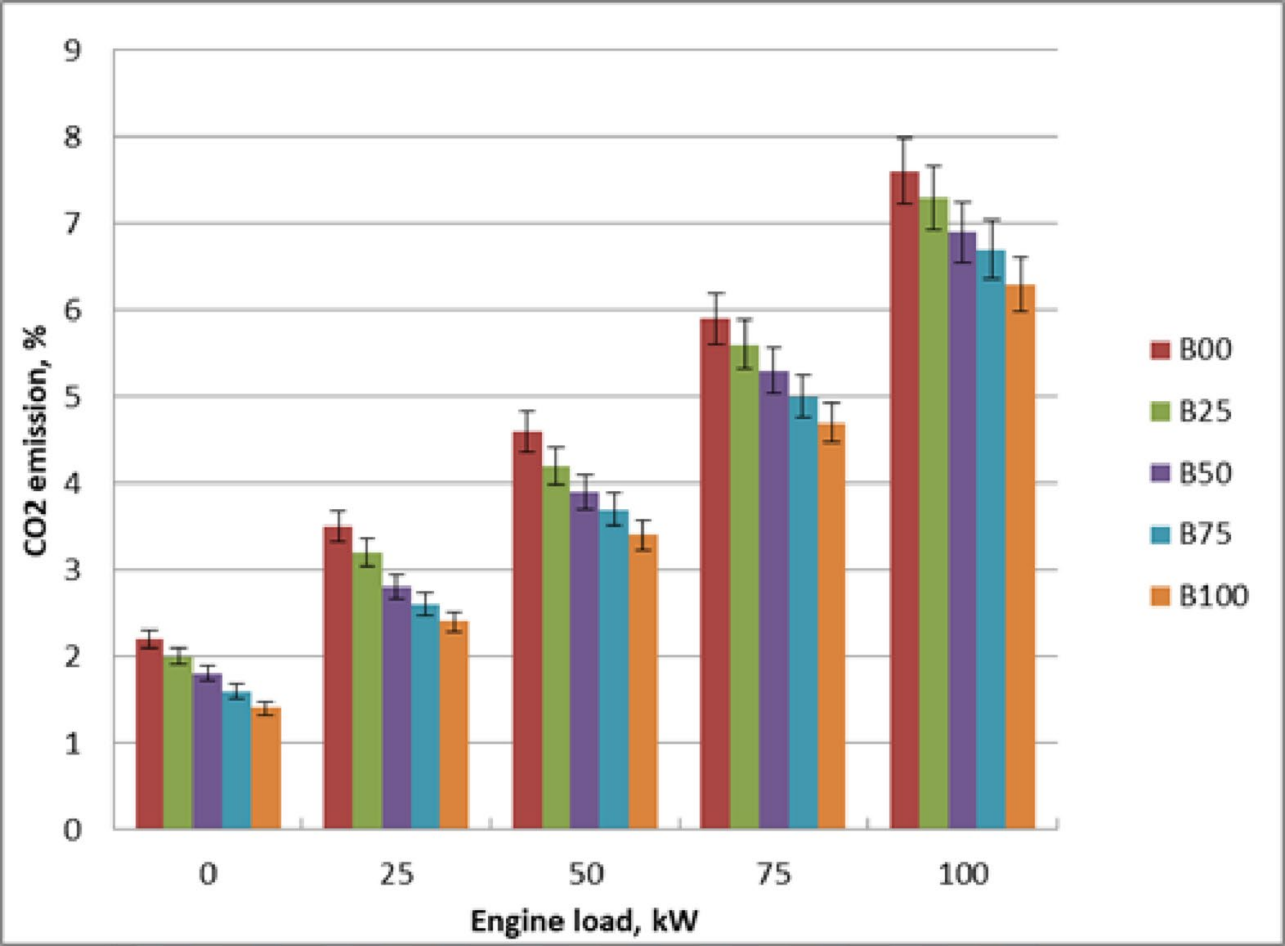
Influence of methyl ester mixtures on CO_2_ at engine load variation.

Figure 13 illustrates how engine load affects carbon dioxide levels for methyl ester mixtures at peak load. An increasing engine load causes the cylinder gas temperature to rise, which raises CO_2_ emissions. Engine load affects carbon dioxide concentrations via altering the gas cylinder’s temperature and converting CO to CO_2_. Increasing the biodiesel mixing ratio lowers CO_2_ emissions since methyl ester has higher oxygen percentage and less carbon than pure diesel. Methyl ester’s oxygen content improves improved combustion and lowers the fuel-rich zone building. Consequently, for the same energy output, less carbon is oxidised, resulting in slightly reduced direct CO₂ emissions. Methyl ester showed improved combustion efficiency compared to diesel oil. Molecular composition of B100 is rich in oxygen, which improves combustion and reduces the likelihood of a fuel-rich zone. Compared to diesel oil, biodiesel decreased carbon dioxide emissions by 20% when operating at full load. The literature validated these findings^9,15,23^.

### NOx emissions

**Fig. 14:**
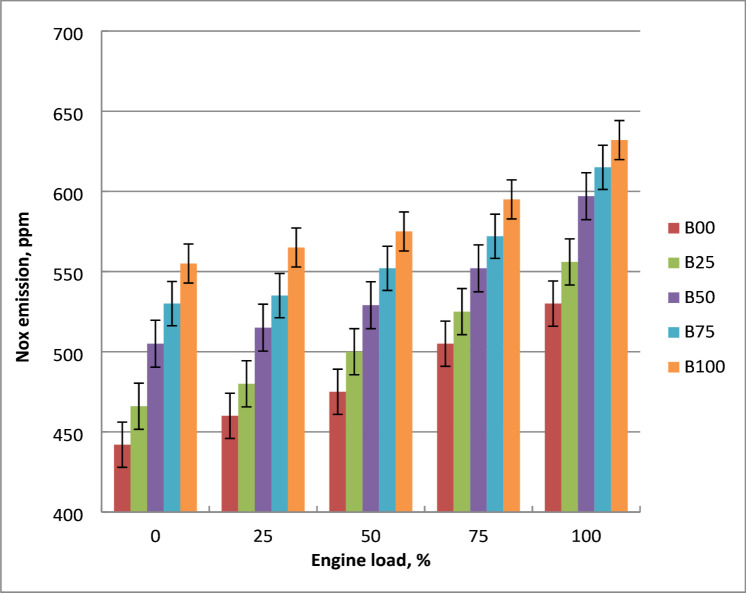
NOx concentrations with brake power change for biodiesel blends.

As shown in Fig. 14, engine output power affects the NOx concentrations from biodiesel blends. Thermal nitrogen oxide production is influenced by temperature, residence time, and cylinder oxygen content. At low engine loads, the fuel-air blend is lean but at high engine loads, it is rich. The rise in cylinder temperature is the reason for the increase in NOx concentrations. As engine load increased, more cylinder turbulence resulted in a richer mixture. At high cylinder combustion temperatures, dissociated nitrogen and oxygen combine to form thermal NOx. Nitrogen oxides concentrations are increased in tandem with the amount of methyl ester. Adiabatic flame temperature rises noticeably as a result of all of this, increasing the NO_x_ emissions of methyl ester relative to pure diesel. The engine cylinder’s increased turbulence creates a richer A/F mixture. These trends are grounded in combustion physics. Biodiesel has a greater cetane number, resulting in an earlier start of combustion and longer residence time at high temperatures. This allows more nitrogen and oxygen to react and generate NOₓ. The rise in NOx with biodiesel concentration, biodiesel NOx paradox’ stems from higher combustion temperatures and advanced combustion timing, both promoting thermal NOx formation. A decrease in ignition delay, air mixing, and fuel preparation time has been blamed for the increase in NOx. Compared to diesel oil, B100 produces 45% increased NOx when running at the highest load. The patterns of NOx emissions were consistent with earlier studies^14,21,23^.

### HC emissions

**Fig. 15:**
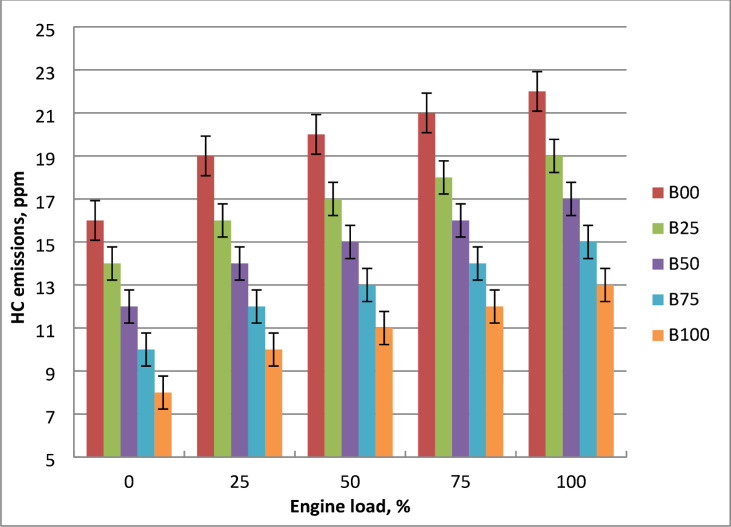
HC concentrations of tested blends at brake power variation.

The link between engine brake power and HC concentrations for diesel and methyl ester blends is shown in Fig. 15. Increased engine loads, cylinder temperatures, and fuel consumption all result in higher hydrocarbon concentrations in methyl ester mixtures. This is because of the high engine brake power, which results in a rich fuel combination and a scarcity of oxygen. At all engine loads, methyl ester lowers HC concentrations due to high oxygen content. When methyl ester is substituted for diesel fuel, it emits lower hydrocarbons due to its higher cetane number. Because biodiesel contains more oxygen, more particles oxidize during diffusion combustion, which improves its capacity to lower HC emissions. The O_2_ in biodiesel facilitates improved combustion and decreases the likelihood of fuel-rich zone production. Higher cetane number of biodiesel result in more controlled combustion, lowering the risk of misfire or incomplete combustion, both of which are primary causes of HC. Methyl ester has superior combustion efficiency over crude diesel because of its higher oxygen content. When using biodiesel instead of diesel fuel when the engine is running at full load, HC emissions are reduced by 43%. Findings of the literature support the trend in HC emissions^7,9^.

### Smoke opacity

**Fig. 16:**
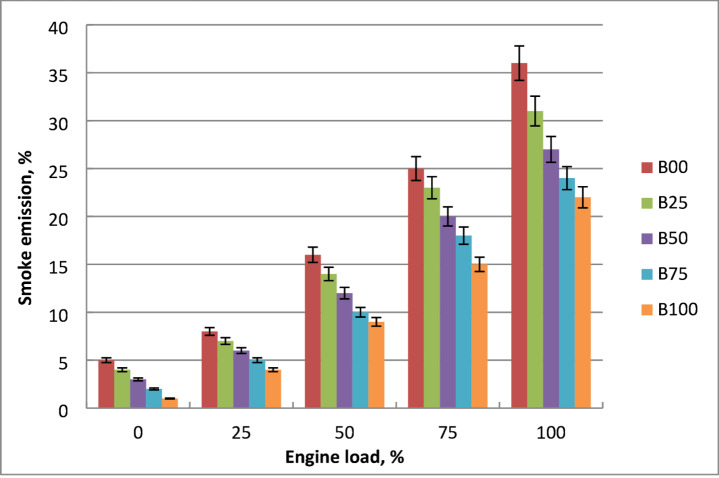
Smoke changes of all fuels at engine output changes.

The influences of engine output power and different biodiesel blends on smoke emissions are depicted in Fig. 16. More smoke is produced since the engine was running faster and using more fuel. At lower engine loads, less smoke is created since there is more oxygen present. Because of the rise in fuel consumption, a drop in oxygen content resulted in observably increased smoke concentrations at high engine output power. The more biodiesel produced the less smoke. Oxygen of methyl ester was the reason for the reduction in smoke. Smoke increased along with fuel usage and output power. Diesel mixtures with methyl ester produced less smoke than those containing ordinary diesel. Biodiesel burns and emits smoke. Biodiesel typically contains low aromatics and sulfur, both of which contribute significantly to soot generation in diesel combustion. Their absence results in cleaner combustion and reduced soot nucleation. Biodiesel is better able to reduce smoke emissions during diffusion combustion by increasing the amount of particle oxidation that takes place. The oxygen in methyl esters increases combustion efficiency, decreases smoke, and enhances ignition. When diesel engine runs at full load, B100 has decreased smoke emission about pure diesel oil by 45%. The findings were confirmed by other studies^15,19,21^.

### Simulation parameters of machine learning models

The simulation parameters of the three models as Multi-Layer Perceptron (MLP), Random Forest, and XGBoos are designed to optimize the prediction performance. Three hidden layers with 50, 20, and 10 neurons each make up the structure for the MLP. MLP activation function is Rectified Linear Unit (ReLU). Also, the Adam optimizer is used with a learning rate equal to 0.001, with early stopping equal to 0.0001. The Random Forest, and XGBoost models use the default configuration parameters.

### Particle swarm optimization

Using the predictions of regression models, Particle Swarm Optimization (PSO) determines the best combination of input variables, such as engine load and blending ratio, to either maximize or reduce a weighted objective function, sometimes indicated the fitness function. This guarantees that the final decision considers all performance metrics (such as efficiency and emissions) based on the established priorities. The efficiency of PSO allows it to quickly identify high-quality solutions without the need to evaluate every possible combination, leading to significant reductions in both time and resource sources.

**Fig. 17:**
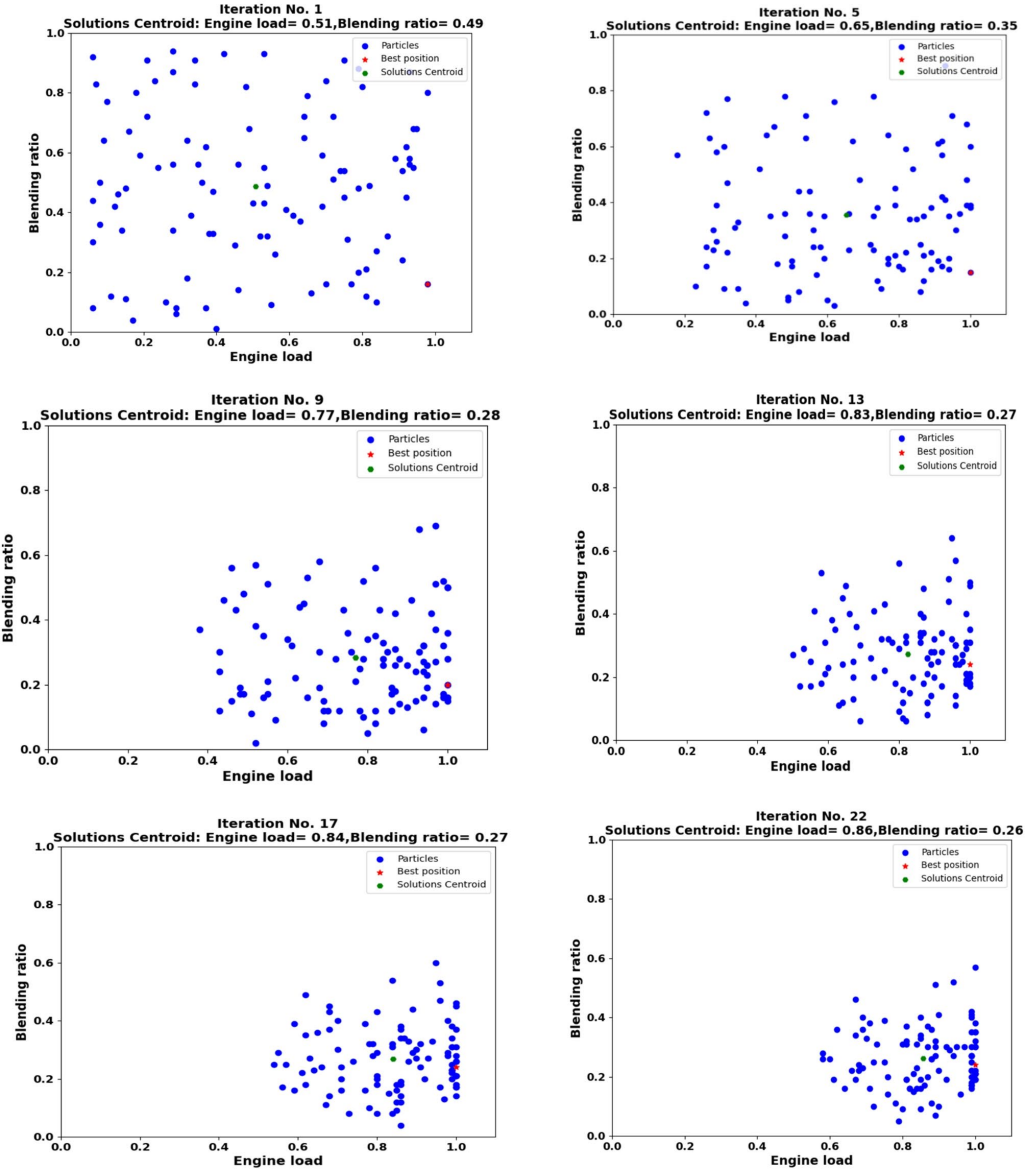
Particle Swarm Optimization convergence sequence showing iterations 1, 5, 9, 13, 17, and 22. Blue dots represent individual particles, red star indicates global best position, and green dot shows swarm centroid.

Figure 17 illustrates the iterative convergence process of the Particle Swarm Optimization (PSO) algorithm toward the optimal combination of engine load and biodiesel blending ratio. The figure consists of a sequence of subplots showing six key iterations (1, 5, 9, 13, 17, and 22) from the total 100 iterations performed, documenting the evolution of the swarm’s search strategy. Each subplot represents the two-dimensional search space defined by engine load (x-axis, 0–100%) and blending ratio (y-axis, B0–B100). The swarm consists of 100 particles (represented by blue dots), each representing a potential solution candidate. The red star indicates the global best position discovered up to that iteration and the solution with the highest fitness value. The green dot shows the centroid (average position) of the entire swarm, indicating the collective tendency of the particles. The optimization process demonstrates a clear convergence pattern. In iteration 1, particles are randomly distributed throughout the search space, with a centroid at approximately (load = 0.51, blend ratio = 0.49), indicating an initial broad exploration phase. As iterations progress, the swarm collectively moves toward regions of higher fitness. By iteration 5, the centroid shifts to (0.65, 0.35), showing a clear preference for higher engine loads and moderate biodiesel blends. This trend continues through iterations 9 and 13, where the centroid reaches (0.77, 0.28) and (0.83, 0.27), respectively, indicating refinement toward specific optimal regions.

**Fig. 18:**
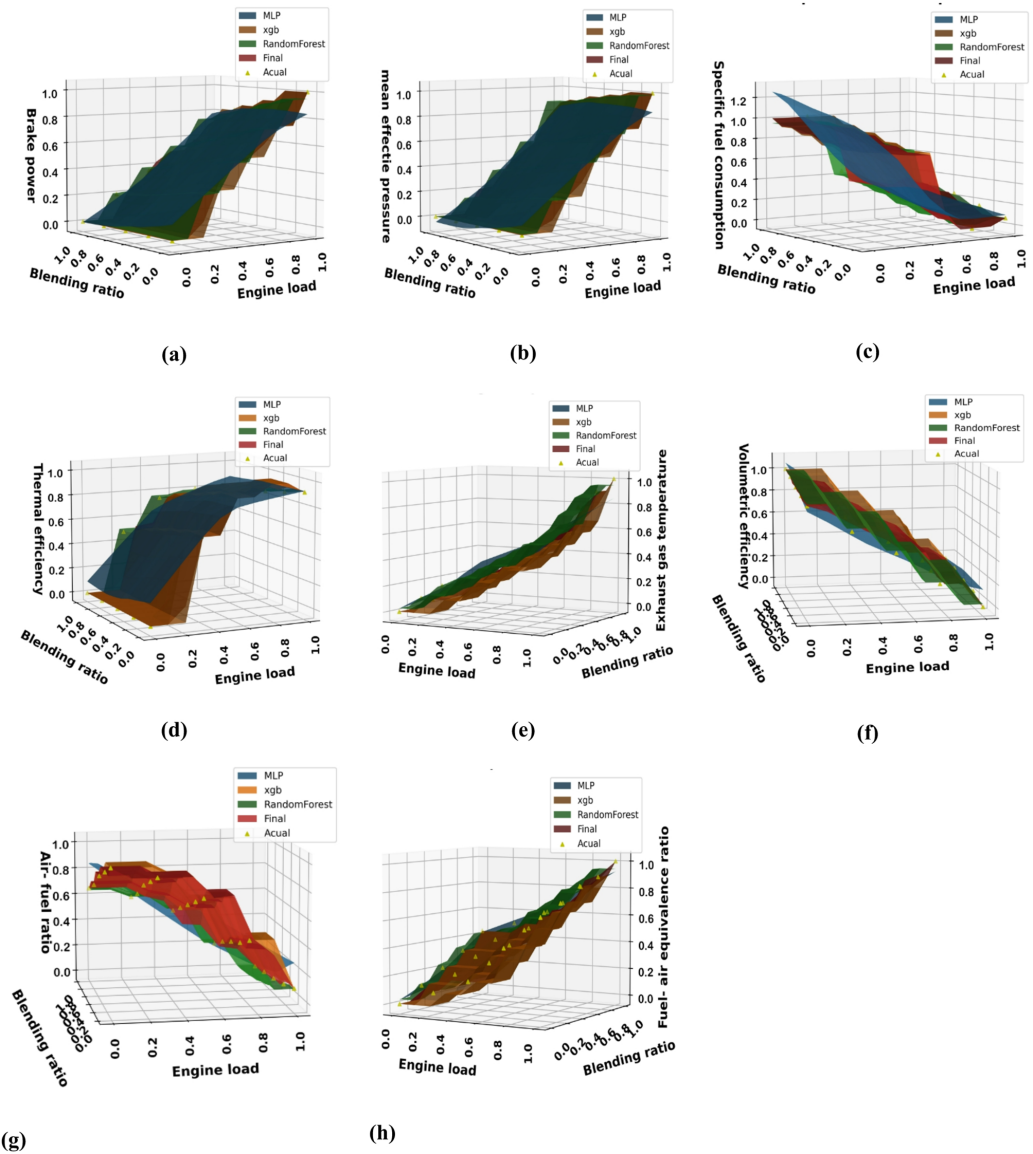
Experimental vs. predicted engine performance: (**a**) Brake power, (**b**) Mean effective pressure, (**c**) BSFC, (**d**) BTE, (**e**) EGT, (**f**) Volumetric efficiency, (**g**) Air-fuel ratio, (**h**) Equivalence ratio.

The convergence becomes particularly evident in iterations 17 and 22, where particles cluster tightly around the global best position at (0.86, 0.26). This spatial concentration demonstrates that the algorithm has successfully identified a robust optimum. The consistent reduction in blending ratio from 0.49 to 0.26 across iterations suggests that moderate biodiesel blends (around B26) combined with high engine loads (around 86%) yield the best compromise between performance and emissions. The dynamic adjustment of velocity ranges from (− 0.05, 0.05) in early iterations to (− 0.01, 0.01) after iteration 10 enables the algorithm to transition effectively from global exploration to local exploitation. This strategic balance ensures thorough search coverage while allowing precise refinement near promising solutions. Figure 18 thus provides visual evidence of the PSO algorithm’s effectiveness in navigating complex multi-objective optimization landscapes, ultimately identifying the optimal operating conditions that maximize the defined fitness function balancing engine performance with emission constraints.

The convergence of the Particle Swarm Optimization algorithm to a specific optimum of 86% engine load with B26 biodiesel blend (Fig. 18) is a finding of significant practical importance. This result is not arbitrary but is a direct consequence of the carefully weighted fitness function, which encoded the real-world objective of balancing performance with environmental concerns. The high optimal load is driven by the positive weighting of brake power and thermal efficiency, which generally improve with load due to reduced relative heat losses and improved combustion efficiency. However, the algorithm correctly avoided the maximum load condition, where the sharp rise in emissions, particularly NOx, would have penalized the fitness score. Concurrently, the identification of a B26 blend as optimal reveals a key trade-off in biodiesel utilization. While high biodiesel blends (like B100) reduce CO, HC, and smoke through more complete combustion, they also significantly increase NOx emissions and fuel consumption. The B26 blend represents an optimal compromise, offering substantial emission reductions over pure diesel without the severe NOx increase and power loss of high-percentage blends. Identifying of B26 as optimal provides actionable insight: mid-level blends can achieve significant emission reductions with minimal performance loss, suggesting very high blends may be inefficient.

### Comparison between developed models and experimental

**Fig. 19:**
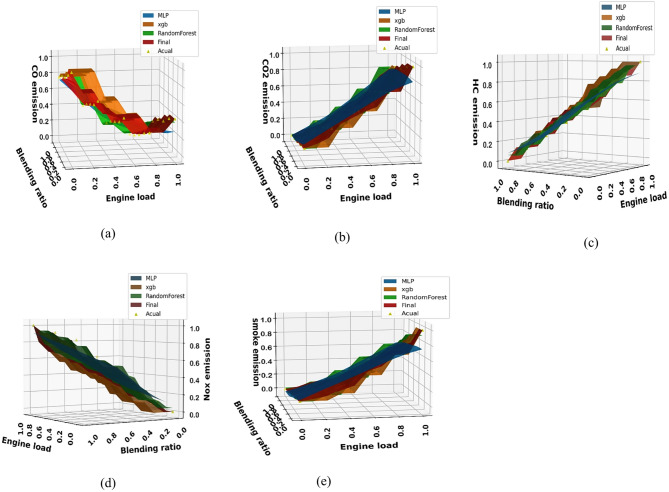
Experimental vs. predicted emissions: (**a**) CO, (**b**) CO_2_, (**c**) HC, (**d**) NOx, (**e**) Smoke opacity.

This section uses XGBoost, random forest, ANN, and hybrid models to forecast the output variables, including brake power, mean effective pressure, BSFC, BTE, EGT, A/F, fuel-air equivalency ratio, CO, HC, NOx, CO_2_, and smoke, using the experimental data that has been provided as shown in Figs. 18 and 19. Figure 18 provides comprehensive visual validation of the predictive models for performance parameters through a series of three-dimensional surface plots that compare experimental measurements with model predictions across the complete operational domain. Each subplot, from (a) Brake power to (h) Equivalence ratio, represents a specific engine performance parameter, with the x-axis showing engine load (0-100%), the y-axis representing biodiesel blending ratio (B0–B100), and the z-axis displaying the corresponding parameter value. The experimental data points, marked as “Actual” in the legends, are distributed across the load-blend space according to the experimental design matrix, representing the ground truth measurements obtained from engine testing.

Similarly, Fig. 19 shows the comparison for emission parameters, with subplots from (a) Carbon monoxide (CO) to (e) Smoke opacity. The prediction surfaces generated by four different modeling approaches as MLP, XGBoost, Random Forest, and the final Hybrid model are overlaid on the same coordinate system, allowing for direct visual comparison between predicted and experimental values. The key observation across all subplots is the consistent close alignment between the Hybrid model surface (labeled “Final”) and the spatial distribution of experimental data points. For instance, in the BTE plot Fig. 18d, the Hybrid model accurately captures the efficiency peak at intermediate load conditions and its variation with blending ratio, closely following the actual measurement points. Similarly, for emission parameters like NOx Fig. 19d and CO Fig. 19a, the Hybrid model surface correctly represents the increasing trends with higher engine loads while maintaining appropriate sensitivity to biodiesel blending effects. The consistent performance across diverse parameter types from power-related metrics like brake power Fig. 18a and mean effective pressure Fig. 18b to complex emission characteristics like HC Fig. 19c and smoke opacity Fig. 19e demonstrates the robustness of the Hybrid modeling approach. Particularly noteworthy is the model’s accuracy in capturing non-linear interactions between engine load and blending ratio, as evidenced by the curved surface contours that faithfully follow the experimental data distributions. This visual evidence, combined with the quantitative metrics, provides strong validation of the Hybrid model’s suitability for engine optimization and emission prediction tasks.

Conversely, the reduction in CO and unburned HC emissions is a direct benefit of the fuel-bound oxygen in biodiesel molecules, which facilitates more complete oxidation, especially in fuel-rich zones within the combustion chamber that are prevalent at high loads. The model’s accurate capture of the non-linear relationship between blending ratio and volumetric efficiency Fig. 18f further underscores its capability to map complex physicochemical interactions, such as the displacement of intake air by biodiesel vapor and the effects of charge cooling.

**Fig. 20:**
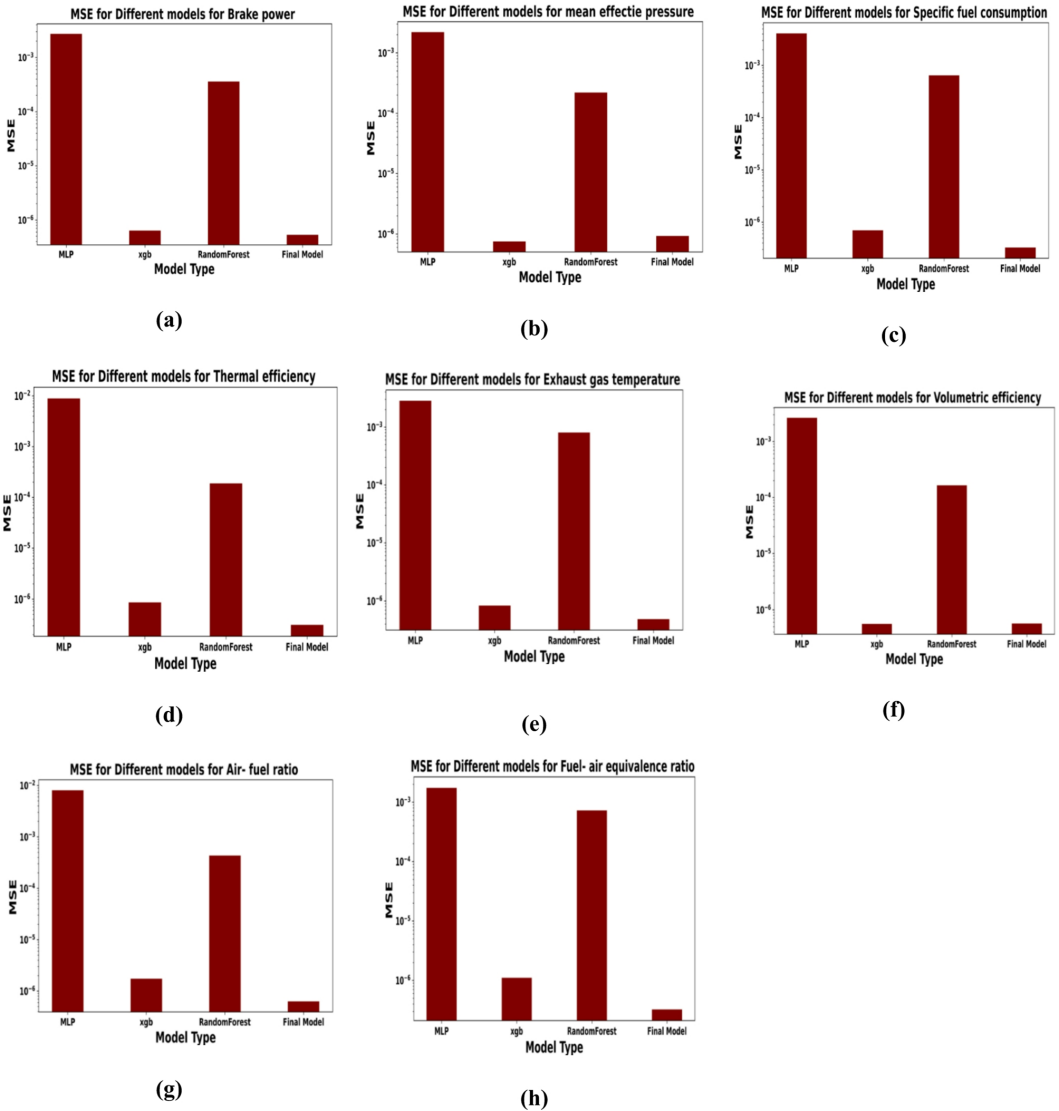
Prediction MSE for performance parameters: (**a**) Brake power, (**b**) Mean effective pressure, (**c**) BSFC, (**d**) BTE, (**e**) EGT, (**f**) Volumetric efficiency, (**g**) Air-fuel ratio, (**h**) Equivalence ratio.

**Fig. 21:**
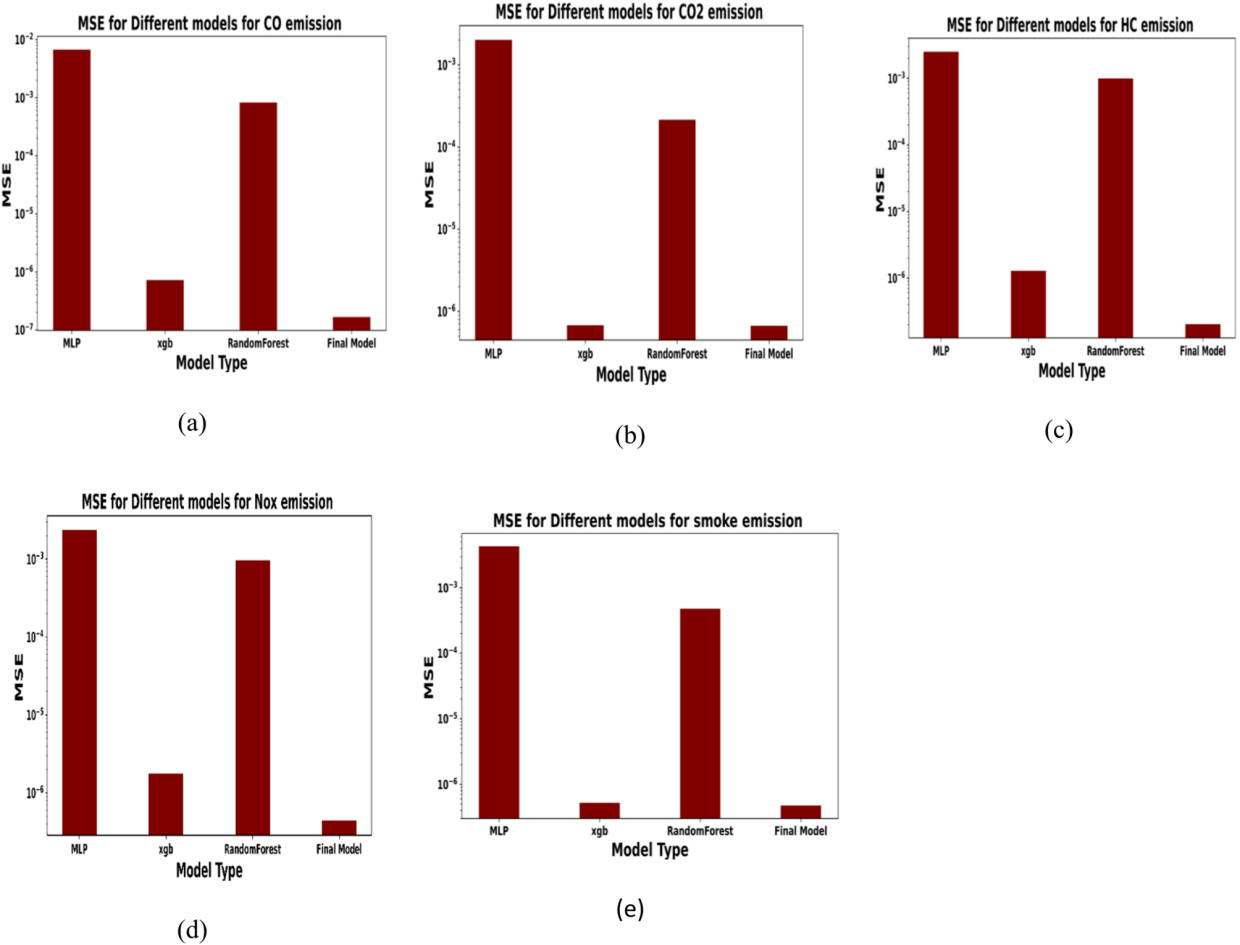
Prediction MSE for emissions: (**a**) CO, (**b**) CO_2_, (**c**) HC, (**d**) NOx, (**e**) Smoke opacity.

The following metrics are used to compare the models. The mean square error (MSE) calculated as:

$$\:MSE\left(y,\:\widehat{y}\right)=\frac{1}{N}\sum\:_{i=1}^{N}{\left({y}_{i}-{\widehat{y}}_{i}\right)}^{2}\:$$

where $\:y\:is\:$measured output is value and $\:\widehat{y}$ is the predicted target value. Examination of Mean Squared Error (MSE) outcomes from the deployed models provides significant insights into their performance attributes as depicted in Figs. 20 and 21. The Multi-Layer Perceptron (MLP) recorded the worst MSE performance across most parameters, such as BTE Fig. 20d and CO emission Fig. 21a. This may be related to experimental data limitation. Conversely, the XGBoost Regressor realized the lowest MSE among the individual models, as clearly seen in subplots like brake power Fig. 20a and BSFC Fig. 20c. XGBoost has the capability in managing non-linear relationships and utilizing its gradient boosting framework to gradually minimize prediction errors. However, precise adjustment of parameters like learning rate, tree depth, and regularization factors is necessary for XGBoost to function well. The Random Forest Regressor showed moderate performance, with an MSE that is lower than that of the MLP but higher than that of XGBoost, evident in parameters like mean effective pressure Fig. 20b and HC emission Fig. 21c. This is consistent with the Random Forest’s method of averaging predictions from independently trained trees, which provides robustness and mitigates variance. Finally, the hybrid model, which integrates predictions from MLP, XGBoost, and Random Forest into meta-features, achieved the lowest overall MSE across all parameters, as quantitatively demonstrated in every subplot of Figs. 20 and 21. This illustrates the benefits of stacking, where the strengths of individual models are combined to offset their weaknesses. While the hybrid model excels in minimizing bias and variance, it also introduces additional computational complexity. In summary, the MSE results for the 13 target parameters emphasize the advantages of ensemble and hybrid methodologies for predictive tasks involving tabular data, while also highlighting the limitations of MLP in this scenario.

The comprehensive results presented demonstrate not only the effectiveness of the proposed hybrid modeling and optimization framework but also provide deep insights into the interplay between engine operation, fuel composition, and performance-emission trade-offs. The superior predictive accuracy of the hybrid stacked ensemble model, as quantitatively validated by its minimal MSE across all sub-figures in Figs. 20 and 21, stems from its unique architecture. The hybrid model acts as a committee of experts, using a meta-learner to intelligently combine the predictions from the MLP, XGBoost, and Random Forest models. This allows it to compensate for the individual weaknesses of each algorithm, particularly the tendency of the standard MLP to overfit on small datasets, thereby achieving a level of robustness and accuracy unachievable by any single model.

Table 2 presents a detailed comparison of the Mean Squared Error (MSE) associated with various engine performance and emission parameters across four models: MLP, XGBoost, Random Forest, and a hybrid model. In the case of brake power, the MSE values indicate a distinct pattern, with MLP exhibiting the highest error (0.002721589), followed by Random Forest (0.000432729). XGBoost shows a significantly lower error (6.38E−07), while the hybrid model achieves the best performance with the lowest error (5.34E−07). A similar trend is observed for mean effective pressure, where MLP again records a high MSE (0.002204502), whereas Random Forest (0.00017572) and XGBoost (7.52E−07) demonstrate improved performance, with the hybrid model yielding the most favorable result (9.25E−07). In the context of emissions predictions, particularly for CO emissions, MLP again registers the highest error (0.006708499), being notably surpassed by XGBoost (7.19E−07) and Random Forest (0.000765264). The hybrid model, however, provides the most precise prediction, achieving an MSE of only 1.66E−07. Regarding thermal efficiency, MLP’s error stands at 0.008889129, this is significantly greater than that of XGBoost (8.64E−07) and Random Forest (0.000185856), while the hybrid model reduces the error to 3.11E−07. For specific fuel consumption, MLP records an error of 0.004083075, whereas XGBoost (7.01E−07), Random Forest (0.000482096), and the hybrid model (3.95E−07) show progressive enhancements. Similarly, the hybrid model achieved the lowest error. Overall, the hybrid model demonstrates superior predictive performance across all metrics, while MLP consistently achieves the worst performance. These findings underscore that the hybrid model, effectively leveraging the strengths of the individual models, has a potential capability in reducing predictive errors.

Our primary goal was to compare the predictive accuracy of four different modeling approaches (MLP, XGBoost, RF, Hybrid) across 13 distinct output variables. MSE is a stringent, widely accepted metric for regression tasks. Its quadratic nature heavily penalizes large errors, making it highly effective for clearly differentiating between models’ performance, especially when the errors are very small, as in our case (ranging from 10^−3^ to 10^−7^). Mean Square Error was chosen as the primary metric because it is sensitive to large deviations and can provide a direct quantitative measure of prediction accuracy during the model training and testing phases. The fitness function for our Particle Swarm Optimization (PSO) was fundamentally based on minimizing prediction error. Using MSE, this is directly related to the error being minimized, ensured consistency throughout our methodology.

**Table 2:** Mean square error (MSE) for MLP, XGBoost, random Forest, and final hybrid model.

Parameters	MLP	XGBoost	Random forest	Hybrid model
Brake power	0.002721589	6.38E−07	0.000432729	5.34E−07
Mean effective pressure	0.002204502	7.52E−07	0.00017572	9.25E−07
BSFC	0.004083075	7.01E−07	0.000482096	3.95E−07
BTE	0.008889129	8.64E−07	0.000185856	3.11E−07
Exhaust gas temperature	0.001735155	1.10E−06	0.000762397	3.24E−07
Air-fuel ratio	0.002840784	8.34E−07	0.00084836	4.86E−07
Equivalence ratio	0.00805232	1.75E−06	0.000523592	5.62E−07
Volumetric efficiency	0.0026497	5.59E−07	0.000187546	5.69E−07
CO emission	0.006708499	7.19E−07	0.000765264	1.66E−07
CO_2_ emission	0.002008877	6.76E−07	0.000309425	6.67E−07
HC emission	0.002500108	1.28E−06	0.000922653	2.03E−07
NOx emission	0.002344321	1.75E−06	0.000894478	4.38E−07
Smoke emission	0.004297152	5.20E−07	0.000448199	4.73E−07

## Conclusions

WCO was used to produce methyl ester in this study, and the properties of the biodiesel blend nearly resemble ASTM diesel. Different ratios of diesel and biodiesel are used, such as 25, 50, 75, and 100%. Models using XGBoost, random forest, MLP and hybrid models are utilized in experimental studies on engine emissions and performance for biodiesel and diesel mixtures. This study demonstrates that significant research novelty can be achieved through the intelligent integration of existing mathematical tools. The proposed hybrid stack and its use within an optimization pipeline where PSO efficiently queries the model to find the best operating conditions offer a novel methodology that transcends the capabilities of its individual components.

The following is a summary of the results obtained:

- Output power and mean effective pressure for B100 are 25 and 24% decreased about pure diesel engine at highest brake power and 1500 rpm, respectively. In relation to crude diesel, biodiesel raised the BSFC, equivalence ratio, and EGT by 28%, 22%, and 23%, respectively, at full engine output power. Biodiesel’s volumetric efficiency and air-fuel ratio are 4% and 15% declined about diesel oil at 100% engine output power, respectively.
- At 1500 rpm engine speed and maximum brake output power, the highest decreases in carbon monoxide, CO_2_, hydrocarbons, and smoke were 12, 13, 44, and 48%, respectively using pure biodiesel in relation to diesel oil. Methyl ester application reduces nitrogen oxides by 23% about diesel oil.
- Hybrid model incorporates MLP, XGBoost, and RF predictions into meta-features, had the lowest total MSE. This demonstrates the advantages of stacking, which combines the advantages of several models to counteract their drawbacks. The hybrid model approach is very good at reducing bias and variation. MSE findings for the 13 target parameters show the limitations of MLP in this situation while also demonstrating the benefits of ensemble and hybrid approaches for prediction tasks using tabular data.
- Using biodiesel blends reduce engine emissions as HC, CO and smoke compared to diesel oil. B100 reduces CO, CO_2_, HC and smoke emissions by 25, 20, 43 and 45% in comparison to diesel fuel. Waste is diverted from disposal by using WCO. Compared to fossil diesel, life cycle GHG emissions are usually lower. When WCO is accessible locally and processing is scaled appropriately, economic viability increases. Particularly at lower blend ratios up to 20%, WCO biodiesel offers engine performance (power, specific fuel consumption, and thermal efficiency that is comparable to diesel. WCO biodiesel’s increased oxygen content improves combustion efficiency. WCO is a cheap and renewable that minimizes pollution from the waste disposal and lessens reliance on fossil fuels. So, WCO-based biodiesel is a viable alternative fuel for CI engines.

This study is a robust methodology for navigating the complex multi-objective optimization landscape of sustainable engine operation, bridging the gap between data-driven modeling and fundamental engine thermodynamics. The main result of this study is the successful development of a hybrid AI and PSO optimization framework, which identified that operating a diesel engine at 86% load with a 26% biodiesel blend (B26) provides the optimal balance between engine performance and emission reduction for WCO biodiesel.

### Hybrid modeling and optimization strengths and weaknesses

In statistical and regression models, simple structure, easy interpretation, and little computational effort. Limited capacity to capture nonlinear and linked effects across variables; accuracy suffers under complex biodiesel or multi-fuel circumstances. ANN and Machine Learning Models have strong nonlinear mapping capabilities, excellent predictive accuracy, and adaptability to big datasets. Training takes a large amount of data; there is a risk of overfitting; and model interpretability is limited. In hybrid and optimization-based models, it combines the strengths of numerous methods to increase accuracy, robustness, and generalization, as well as the ability to tune and optimize parameters. It is computationally expensive and requires meticulous parameter selection and validation to assure dependability. The hybrid XGBoost-RF-MLP model optimized by PSO was created to combine the nonlinear learning capability of ensemble and deep models with the global search efficiency of metaheuristic optimization, overcoming the limits of individual methods.

### Limitations and practical implications

While this study establishes a robust methodological framework, its findings should be considered in light of certain limitations. The analysis is based on a constrained experimental dataset from a single engine configuration operating at steady-state conditions. Consequently, the model’s generalizability to other engine sizes, designs, and transient operating cycles remains to be fully validated. Furthermore, the optimization was conducted using a limited set of input parameters, excluding variables such as injection timing. NOx mitigation solutions should be considered in biodiesel operations. EGR systems to lower combustion temperatures, selective catalytic reduction (SCR) and oxidation catalysts to reduce tailpipe NOx, and fuel-borne additives (cerium oxide, titanium dioxide nanoparticles, and water emulsions) to improve combustion and suppress peak temperature are now recognized as effective approaches to reducing NOx emissions in biodiesel-fueled CI engines. The created hybrid ML-PSO framework may significantly minimize the number of expensive and time-consuming engine experiments by accurately projecting performance and emission outcomes for diverse biodiesel-HHO-nanoparticle combinations. Furthermore, the model can guide appropriate blend and additive selection under a variety of load and speed conditions, hence facilitating data-driven optimization and sustainable fuel formulation for CI engines.

### Future work

The dataset will be increased by conducting additional tests and integrating publically accessible engine performance data to improve the hybrid model’s forecasting accuracy. EGR and selective catalytic reduction systems will be used to lower tailpipe NOx. Expanding operational scope will be used to validate the framework under transient engine operations, variable load/speed settings and real-world driving cycles to to prove the hybrid model prediction framework’s robustness and adaptability to realistic engine environments. Exploring fuel variability will investigate the impact of different WCO feedstocks and fuel additives on the model’s predictions and the identified optimum. Exploration of nano additives will improve combustion efficiency and emissions. Combining WCO biodiesel with hydrogen or HHO enrichment will produce nearly zero emissions. Future study will concentrate on incorporating economic feasibility, energy return on investment and environmental impact indicators as carbon footprint into the modeling framework. This integration will allow for a full assessment of WCO biodiesel’s competitiveness against conventional diesel and alternative renewable fuels, offering data-driven insights for policy formulation and large-scale implementation. Generalizability can be assessed by testing the proposed methodology on a wider range of engine sizes and types to confirm its robustness and transferability. The framework will be extended to include multi-fuel (Jatropha, palm, algal biodiesel) and multi-engine datasets, allowing the model to capture broader operational behaviors and increase generalization. This update will also support transfer learning and meta-model adaptation, improving prediction accuracy across a variety of combustion systems and fuel attributes.

## Data Availability

All data generated or analyzed during this study are included in this published article.
